# Cyclic Peptide–Polymer Conjugate Characterization Using 193 nm Ultraviolet Photodissociation Tandem Mass Spectrometry

**DOI:** 10.1021/acs.analchem.5c03375

**Published:** 2026-02-05

**Authors:** Tomos E. Morgan, Alina Theisen, Sean Ellacott, Anisha Haris, Christopher A. Wootton, Julia Y. Rho, Mark P. Barrow, Anthony W. T. Bristow, Sébastien Perrier, Peter B. O’Connor

**Affiliations:** † Department of Chemistry, 2707 University of Warwick, Coventry CV4 7AL, U.K.; ‡ Chemical Development, Pharmaceutical Technology & Development, Operations, AstraZeneca, Macclesfield SK10 2NA, Cheshire, U.K.; § Warwick Medical School, University of Warwick, Coventry CV4 7AL, U.K.; ∥ Faculty of Pharmacy and Pharmaceutical Sciences, Monash University, 381 Royal Parade, Parkville, Victoria 3052, Australia

## Abstract

Cyclic peptide–polymer conjugates offer a unique biocompatible system with many advantages but come at the cost of being analytically challenging. Developing further analytical techniques of complex polymer-conjugate systems is key to understanding synthetic and medicinal properties. In this contribution, a synthetic cyclic peptide–polymer conjugate is analyzed using electron capture dissociation (ECD), infrared multiphoton absorption dissociation (IRMPD), and 193 nm ultraviolet photodissociation (UVPD) on the same mass spectrometry system. IRMPD and UVPD were shown to effectively characterize unconjugated cyclic peptide species. ECD was less informative during cyclic peptide analysis due to the production of multiple sequence scrambling fragments and radical side chain losses. ECD was shown to produce extensive fragmentation and enable the characterization of conjugated side chains of cyclic species. ECD and IRMPD thus provided complementary data, enabling the target analysis of conjugated systems. UVPD effectively characterized both the cyclic peptide and the conjugating polymer in one experiment, being able to produce complete cyclic peptide fragmentation via *b*/*y* fragment pathways and polymer fragmentation via *a*/*x* poly­(2-ethyl-2-oxazoline) fragment pathways.

## Graphical abstract

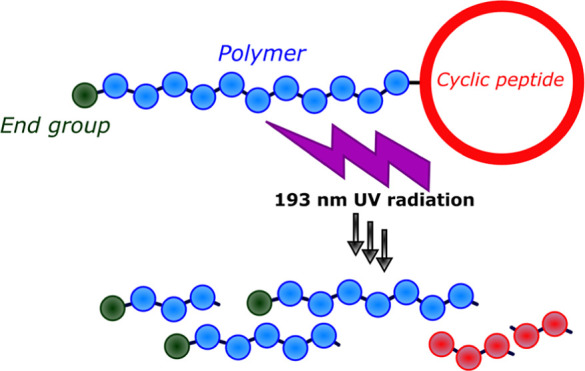

With increasingly potent small molecules being developed for medicinal applications, there is a need for increasingly complex drug delivery vectors.^1^ The properties of the drug delivery vectors can be tuned to greatly increase the efficacy of a drug by taking advantage of biological phenomena such as the enhanced permeability and retention (EPR) effect.^2,3^ Self-assembling nanotubes formed from cyclic peptides produce controlled tubes with specified internal diameters,^4^ and conjugation of polymers to the cyclic peptide can offer a further control of the self-assembly mechanism.^5,6^ Variations in the conjugated polymers can produce thermoresponsive,^7^ pH-responsive,^8–10^ redox-responsive,^11^ and even hydrogel-forming^12,13^ nanotubes for use as drug delivery vectors.^14^ The use of alternating d- and l-amino acids has been shown to produce an amino acid system that can interlink to form nanotube structures though intermolecular bonds; conjugating polymers onto the central cyclic peptide structures can produce controllable nanotube lengths.^15^

Both the cyclic peptide central nanotube and the conjugated polymer can be varied,^16^ offering a unique analytical challenge due to dispersity in the polymer chain and modification of the cyclic-peptide core. Analysis is often carried out qualitatively by nuclear magnetic resonance (NMR) to confirm the presence of the cyclic peptide among polymer signals. Analysis of the final nanotube structure has been carried out by light scattering methods.^6^ NMR and light scattering techniques, though powerful, do not provide the polymer or peptide sequence information that tandem mass spectrometry can provide. MS also adds a level of specificity, separating mixed populations from one another within the same analysis.

Protein–polymer conjugate species have been investigated with the use of mass spectrometry analysis,^17,18^ with peptide–polymer conjugate analysis being carried out by matrix-assisted laser desorption/ionization (MALDI) and electrospray ionization (ESI) coupled to ion mobility MS.^19^ The ability to accurately identify the masses of more complex polymer-conjugate species is vital in confirming that successful synthesis of target delivery vectors has occurred without modification during the conjugation process. Assigning complex MS polymer spectra has been made more facile with the use of Kendrick^20^ mass defect techniques for polymer assignment.^21–23^

Infrared multiphoton dissociation (IRMPD), ultraviolet photodissociation (UVPD), and electron capture dissociation (ECD) mass spectrometry techniques each offer complementary fragmentation methods for biopharmaceuticals.^24–27^ IRMPD produces ergodic vibrational fragmentation via infrared radiation.^28–30^ Radical-based fragmentation via ECD can generate complementary fragments to IRMPD.^31–33^ Within protein MS/MS, the IRMPD fragmentation manifests as fragmentation of the amide bond (*b*/*y*) through fragmentation of the carbon to nitrogen bond. In ECD, fragmentation occurs through the nitrogen to carbon bond α to the amide bond (*c*/*z*).

UVPD offers a unique fragmentation via two dissociation pathways: direct dissociation resulting in electronic excitation or relaxation into a dissociative orbital and internal conversion, where photon energy is converted into vibrational modes and fragmentation occurs in the ground state. Hence, the fragments produced will be like those generated by IRMPD.^34^

Tandem mass spectrometry of various poly­(oxazoline) species has been carried out previously by collisionally activated dissociation (CAD/CID)^35,36^ and radical-based ECD.^37–39^ Recently, interest has grown regarding the use of UVPD methods due to advances in the available laser and MS instrumentation.^40,41^ UVPD has so far demonstrated promising results in the analysis of numerous biomolecules such as peptides,^42,43^ proteins by top-down,^44–46^ and in native top-down experiments,^47,48^ lipids,^49–51^ oligosaccharides,^52^ and nucleic acids.^53^ Depending on the wavelength used, single laser pulse is often sufficient to cause dissociation, allowing very rapid fragmentation and analysis.^54^ Modification of analytes to include a chromophore can allow or enhance fragmentation by UVPD, especially when using longer wavelengths such as 266 nm.^55–57^

Sequence elucidation of cyclic peptides has long been studied and shown to be consistently challenging due to the lack of a defined terminus groups and extensive side reactions caused by fragmentation.^58,59^ All observed cyclic peptide fragment peaks must be formed via secondary dissociation events, as a single dissociation event will break the cyclic peptide but not produce an observable *m*/*z* change. ECD techniques have been shown to provide limited and varying characterization of cyclic peptides due to possible sequence scrambling caused by events such as the free radical cascade.^60^ Cyclic peptides have been analyzed by UVPD showing high cleavage coverage and effective peptide characterization.^61,62^

In this contribution, a poly­(2-ethyl-2-oxazoline) conjugated to an alternating d- and l-amino acid cyclic peptide was analyzed by IRMPD, ECD, and UVPD to compare the effectiveness of fragmentation methods in characterizing both the peptide and the conjugating polymer.

## Experimental Section

**Cyclic peptide–polymer conjugate synthesis:** cationic ring opening polymerization of 2-ethyl oxazoline was carried out, producing a hydroxyl-capped poly­(2-ethyl-2-oxazoline), as described in the Supporting Information. The resulting poly­(2-ethyl-2-oxazoline) hydroxyl was then conjugated onto the cyclic peptide. A full synthetic procedure is given in the Supporting Information.

**Poly­(2-ethyl-2-oxazoline) ethyl xanthate:** cationic ring opening polymerization of 2-ethyl oxazoline was carried out andend capped with potassium ethyl xanthate.

The cyclic peptide–polymer conjugate sample was dissolved into a 99.5% solution of purified water obtained from a Direct-Q3 Ultrapure Water System (Millipore, Lutterworth, United Kingdom) at 20 μM in 0.5% formic acid (Sigma-Aldrich, Dorset, United Kingdom).

All experiments were performed on a 12 T solariX Fourier transform ion cyclotron resonance mass spectrometer (Bruker Daltonik, GmbH, Bremen, Germany) using nanoelectrospray (nESI). Ionization was carried out in positive mode with homemade glass emitters, and all samples were solvated with 50% acetonitrile in 0.1% formic acid. The ionization voltage was optimized for each sample; each varied between 700 and 1100 V. Isolation windows were adjusted for the precursors with *m*/*z* 3 for cyclic peptides and *m*/*z* 5 for the polymer and cyclic peptide–polymer conjugates. Ion accumulation was varied for each precursor, with accumulation for the cyclic peptide–polymer conjugate precursor for UVPD analysis being the most at 7 s. UVPD dissociation was carried out with one or two 6 mJ laser pulses (measured at laser head) from a 193 nm excimer laser (500 Hz, ExciStart XS, Coherent), which in the used setup translated to <0.6 mJ at in the ICR cell.^63^ ECD was carried out using the hollow dispenser cathode operated at 1.5 A, where the ECD bias voltage was set at 1.2 V for the analysis of both the cyclic peptide and the cyclic peptide–polymer conjugate species. Pulse sequences for ECD were controlled as standard within the solariX software; UV laser triggering was triggered through solariX NICE electronics described previously.^63^ UV laser control was carried out with an NI PCI card and driven by an in-house LabView program controlling lasers shots, repetition rate, and timing of UV shots.^63^

The ECD pulse length was lower for the peptide-conjugate due to the higher ion charge compared to that for the cyclic peptide; pulse lengths used were 100 and 300 ms, respectively. IRMPD was achieved by coalignment of a 10.6 μm CO_2_ laser (Synrad J-42, Novanta Inc., Bedford US), 25W continuous wave IR laser with the ICR cell, and UVPD laser described above (Scheme 1). IRMPD analysis of the cyclic peptide was carried out at 45% laser power with a 150 ms pulse for the cyclic peptide and a 250 ms pulse for the cyclic peptide–polymer conjugate.

**Scheme 1:**
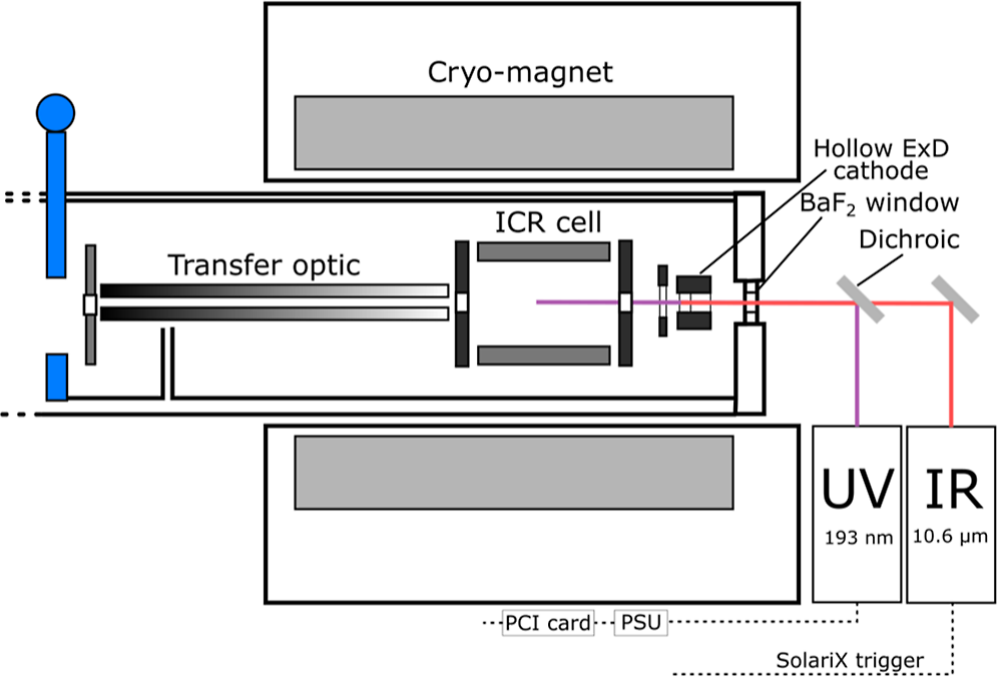
solariX ICR Cell Schematic Showing Modification of the solariX with a UVPD Laser and a BaF_2_ Window, Which Is Transparent to Both IR and UV Radiation

Fourier transform-ion cyclotron resonance (FT-ICR) MS detection used an *m*/*z* range of 98 to 3000 and 4 mega-word (2^22^, 22 bit) digitized data points (length 1.12 s), achieving a magnitude mode resolving power of approximately 250,000 at *m*/*z* 400. All mass spectra were calibrated internally using fragments present in each of the spectra. The peaks used for internal calibration were crosschecked using both the *a* and *x* fragment series to reduce the likelihood of systematic error in calibration. Intact mass spectra were calibrated internally using a generated peak list of expected cyclic-peptide conjugate *m*/*z* values. The Bruker SNAP algorithm was used for peak picking, with the poly­(2-ethyl-2-oxazoline) monomer used as the repeat unit (C_5_H_9_NO). SNAP matches a calculated isotope distribution adjusted to a repeat unit with increasing mass.^64–66^ All fragments were assigned manually with the aid of MKMD plots.

## Results and Discussion

Previous studies into the analysis of poly­(2-ethyl-2-oxazoline) ethyl xanthate polymer species by ECD fragmentation has shown that effective sequence and terminus coverage can be achieved.^37,38^ The observed fragmentation cleavage diagram is presented in Scheme 2, with ECD producing *a*/*x* fragments that are chemically equivalent to *c*/*z* fragmentation in protein ECD MS/MS analysis.

**Scheme 2:**
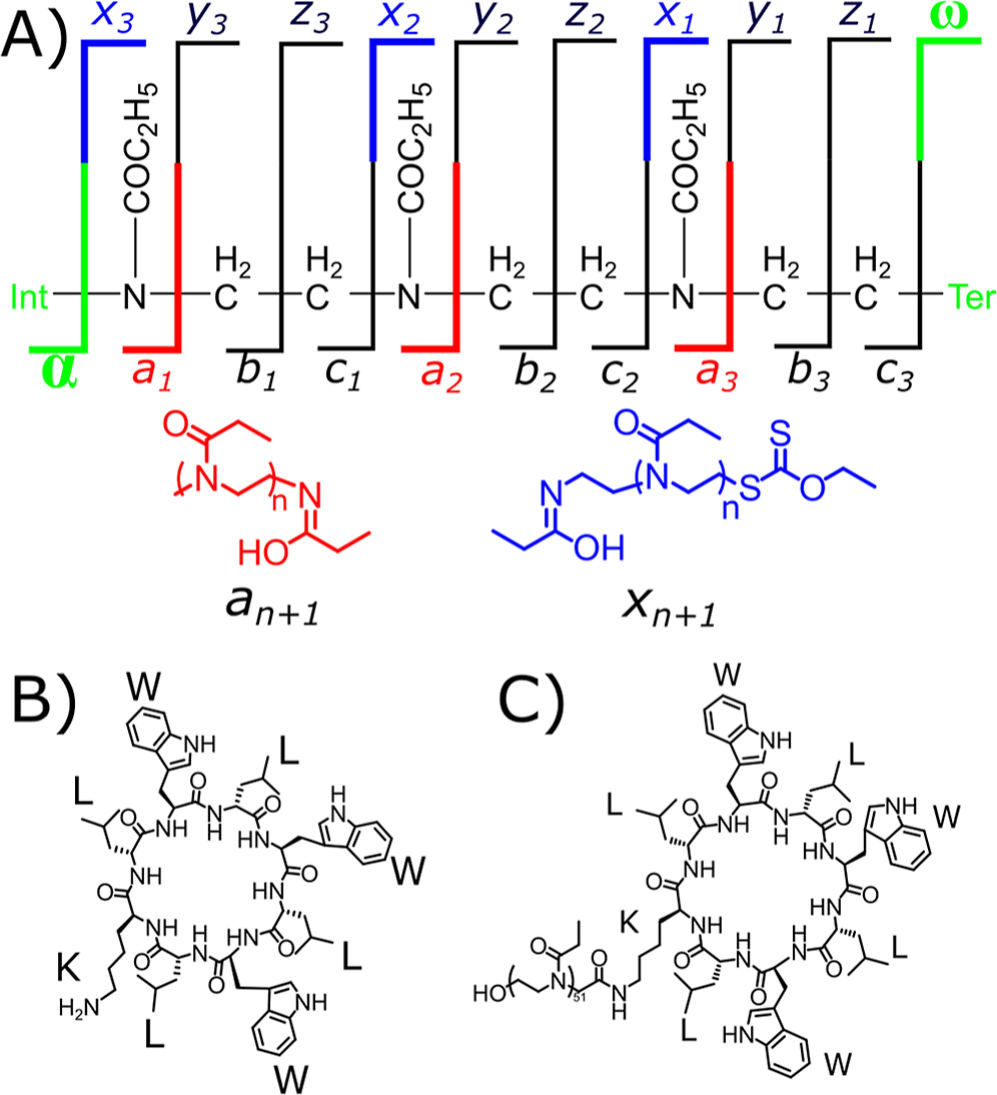
Cleavage Diagram of a Poly­(2-ethyl-2-oxazoline) Ethyl Xanthate: (A) Expected *a*-Series and *x*-Series Fragments Produced from a Homopolymer and Markings of the Side Chain Loss (*b*/*y* Peptide Equivalent) and *a*/*x* Fragments That Are the *c*/*z* Peptide/Protein Fragment Equivalent; (B) Cyclic-Peptide Core Prior to Conjugation; and (C) Poly­(2-ethyl-2-oxazoline) Ethyl Xanthate Conjugated Cyclic Peptide

nESI of the poly­(2-ethyl-2-oxazoline) ethyl xanthate produced mainly +2 and +3 protonated species with a detected monomer range of 11 to 33 monomer units, of which a triply protonated ion at *m*/*z* 672.14, corresponding to the 20-repeat unit hydroxyl terminated polyoxazoline, was isolated for fragmentation. UVPD fragmentation of the polyoxazoline species produced +1, +2, and +3 fragment ions. Both *a* and *x* fragments containing terminal end group species were present. Singly protonated fragments were present from *a*_2_ (*m*/*z* 187.14, 0.1 ppm) to *a*_11_ (1078.76 *m*/*z*, −0.6 ppm); doubly protonated fragments were present from *a*_9_ (*m*/*z* 440.82, −0.1 ppm) to *a*_18_ (*m*/*z* 936.16, 0.5 ppm). No triply charged *a* series fragments were observed in the spectrum.

Figure 1A shows that the ethyl xanthate (S_2_OC_3_H_5_) terminated *x* fragments produced by UVPD were observed as +1, +2, and +3 charge species. UVPD was carried out with a single shot per MS/MS event at 6 mJ of energy/shot. Singly charged fragment series were detected from *x*_2_ (*m*/*z* 321.13, 0.3 ppm) to *x*_11_ (*m*/*z* 1212.75, 0.3 ppm), and doubly charged fragments began at *x*_9_ (*m*/*z* 507.81, 0.3 ppm) to *x*_18_ (*m*/*z* 953.62, 0.1 ppm). Three triply charged *x* fragments were observed at *m*/*z* 636.08, *m*/*z* 669.10, and *m*/*z* 702.13: the *x*_18_, *x*_19_, and *x*_20_ fragments, respectively. The charge was evenly distributed across the polymer as the *a*- and *x*-fragment series produced similar charge distributions. Both polymer termini are characterized by this method.

**Figure 1:**
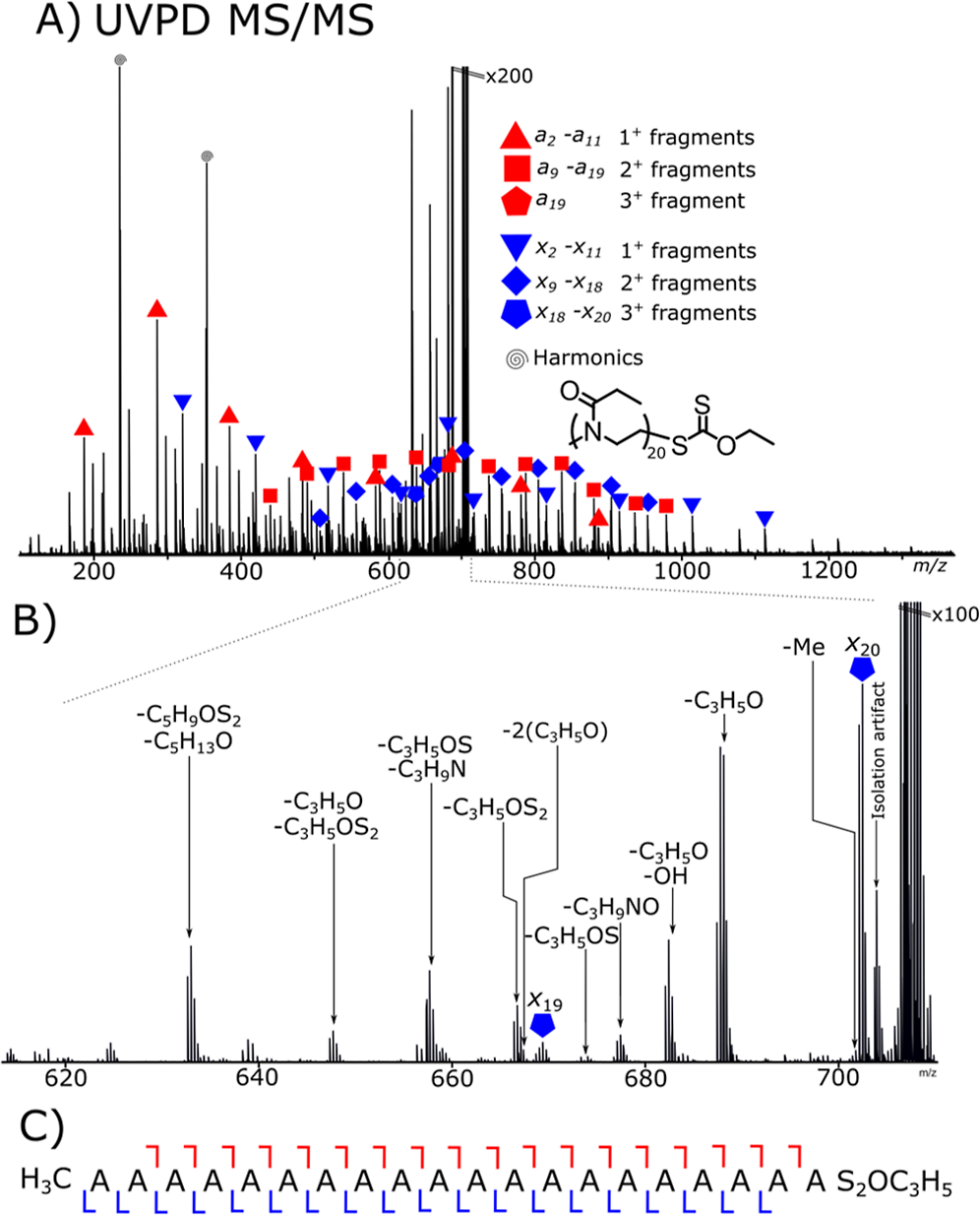
(A) UVPD fragmentation of a triply protonated poly­(2-ethyl-2-oxazoline) ethyl xanthate species. Both *a*- and *x*-fragment series are observed, with multiple neutral losses from the precursor. (B) Triply charged fragments observed, small molecule and side chain losses being the most common from the UVPD event. (C) Fragmentation map showing the total fragmentation coverage.

Overall, the UVPD fragmentation coverage of the polymer was high, with 83% of total possible backbone cleavages being observed. The coverage was very similar to ECD coverage shown in the pervious study.^67^ Calculation of the fragmentation efficiency was carried out by comparison of the fragment peak area to the total peak area of the spectrum. Single shot UVPD fragmentation efficiency was 4% when accounting for all fragmentation peaks including internal fragments and 2% when accounting for just the *a* and *x* fragmentation %.

Figure 1B shows observed neutral losses from the isolated precursor ion similar to *b*/*y* fragmentation events, which results in the breakage of the amide bonds and the loss of a C_3_H_5_O group. C_3_H_5_O loss produced an intense triply charged fragment (*m*/*z* 687.78, C_101_H_183_N_20_O_20_S_2_H_3_^+^, −1.4 ppm), a fragment equivalent to *b*/*y* fragmentation in proteins. Multiple losses are seen with a fragment ion present representing two C_3_H_5_O losses (*m*/*z* = 668.77, C_98_H_178_N_20_O_19_S_2_[H^+^]_3_, 0.5 ppm). Examples of sulfur to carbon bond fragmentation had occurred with a fragment at *m*/*z* 677.13 (C_101_H_183_N_20_O_20_S_1_[H^+^]_3_, −0.4 ppm).

The neutral losses from the precursor can be used to identify the side chains of the polymer as well as the termini. Generally, internal fragments of homopolymers in MS/MS are not chemically/analytically useful, as they do not offer discrete terminal information and provide no useful sequence information.

Tandem mass spectrometry analysis of the cyclic peptide required multiple fragmentation events to occur for fragments to be observed, once to break the cyclic structure and another to form the observable fragment.

The cyclic peptide analysis produces a dense fragmentation spectrum. Cyclic peptide fragmentation relies on two fragmentation events: the first to open the ring and the second to produce a detectable linear fragment peptide. Two fragmentation events added significant complexity to the tandem mass spectrum, first, the presence of fragments starting from any amino acid on the cyclic peptide, and second, the increased fragmentation energy that was needed for two fragment events greatly increased the possibility of neutral losses. Both fragments have two fragment termini as two fragmentation events need to occur and both fragment termini have a different fragment type.

IRMPD analysis of the cyclic peptide produced a fragmentation spectrum, Figure 2A, and the most intense fragment peaks corresponded to two *b*/*y* fragmentation events, with both fragment events occurring at the amide bond. IRMPD fragmentation was tuned so fragments were present and approximately 10% of precursor ions remained compared to the nonfragmentation spectrum. It is clear by the fragment coverage that the most intense fragment series follow the expected fragment ladder. Low levels of fragments (<1%) can be observed that correspond to an inconsistent sequence, e.g., WW, produced by sequence scrambling.

**Figure 2:**
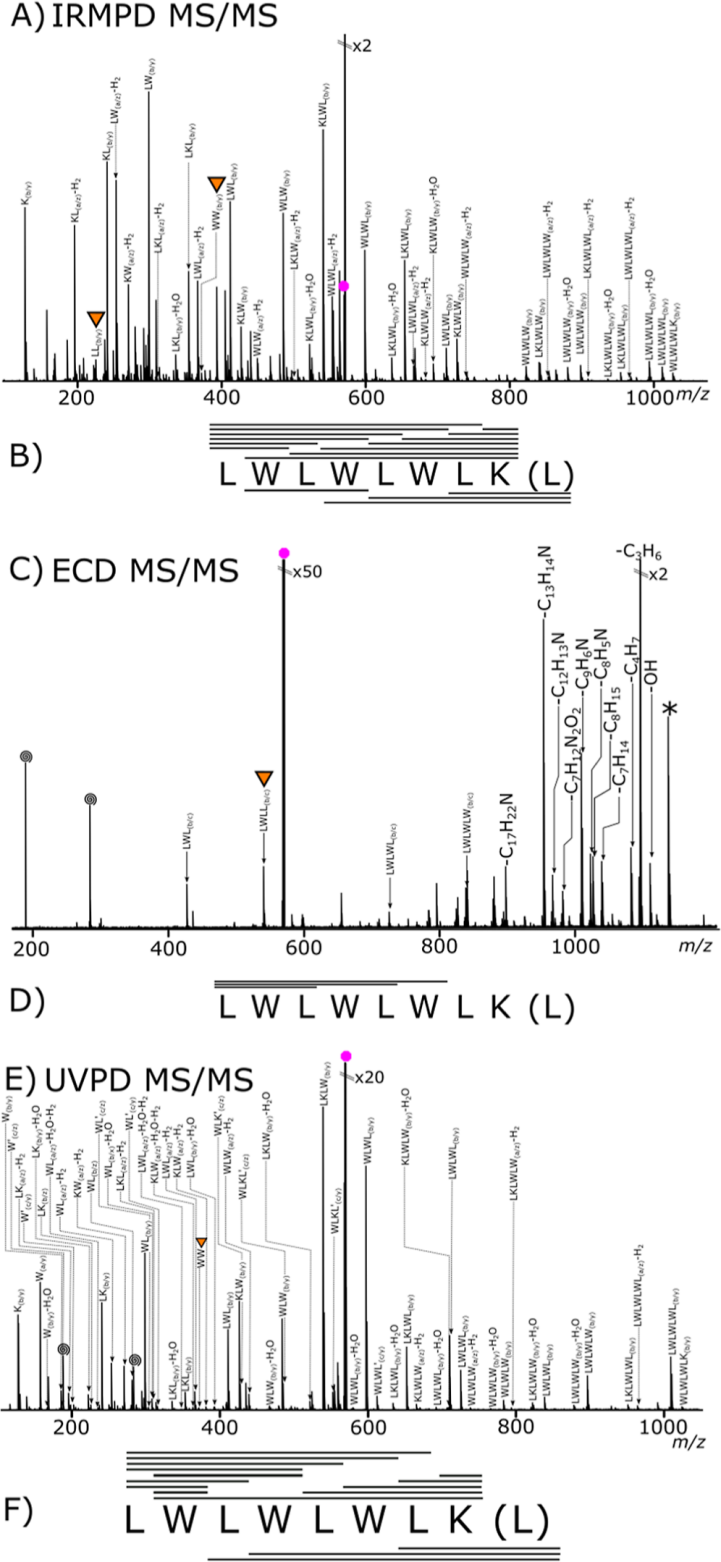
Tandem mass spectrometry analysis of a doubly charged cyclic peptide (structure Scheme 2B): (A) IRMPD MS/MS spectrum and the (B) corresponding fragment coverage lines above the sequence represent detected fragment ions, the “(L)” considers the possibility of fragmentation at another point on the cyclic peptide backbone generating a different sequence initiation points represent peptide fragment coverage detected the “(L)” takes into account the possibility of fragmentation at another point on the cyclic peptide backbone. (C) ECD MS/MS spectrum and the corresponding fragmentation map (D). Inverted triangles highlight peptides with scrambled sequences. (E) UVPD fragmentation spectrum, with corresponding fragmentation map coverage (F). Inverted triangles highlight peptides with scrambled sequences. All spectrum are the result of 50 summed scans.

Most fragments exhibited neutral losses, with the loss of H_2_O being the most prominent loss. The intensity of the H_2_O loss peak compared to that of the same corresponding fragment before water loss showed a large variation from approximately 10% of the intensity to around 80% intensity. Overall, taking the average intensity of H_2_O loss compared to the corresponding fragment, the average H_2_O loss intensity was 35%.

Fragments present in the IRMPD correspond to CONH_3_ loss, equivalent to *a* and *z* fragments. It is not proposed that observation of *a* and *z* fragments in the IRMPD spectrum is due to radical-based primary fragmentation but likely secondary fragmentation and rearrangement due to the increased energy and multiple fragmentation events occurring.^68,69^ The analysis of secondary fragmentation required for cyclic peptide analysis also greatly increases the possibility of isobaric and even isomeric overlap of fragments, making the analysis more complex.

The complexity of the fragment peptide spectra, even for a mostly symmetrical cyclic peptide, such as that presented here, shows the importance of high resolution and mass accuracy for cyclic peptide tandem mass spectrometry. It is worth noting that there is little observation of consistent CO or NH_3_ loss separately, so they may be removed in a concerted process.

ECD analysis of the cyclic peptide, Figure 2C, produced expected results;^60^ low fragmentation coverage was observed and there were a large number of side chain losses. ECD fragmentation was optimized until fragments were observed and the first charge reduced species (CRS) was present at a maximal intensity. Tryptophan side group neutral losses were observed at high abundance. Other neutral losses consist of alkyl chains likely due to the presence of multiple leucine groups and radical rearrangement and loss within these groups. Overall, the ECD experiment provided little analytical information regarding the cyclic peptide species, especially when compared with IRMPD and UVPD data collected herein.

The UVPD fragmentation spectrum, Figure 2E, gave results very similar to those of the IRMPD data, with sequence coverage being achieved through the cyclic peptide. In the UVPD mass spectrum of the cyclic peptide and cyclic peptide–polymer conjugate fragment ions that align with *a*, *b*, *c*, and *x*, *y*, *z* fragments are observed. The most intense fragments observed were that of the *a b* and *a y* fragmentation, constituting approximately 90% of the detected ion peak intensity. Other fragments were also observed; *a* and *z* are common, making up approximately 6% of the remaining detected fragment ion intensity, with *c* and *x* fragments being observed but least common overall. Similar to the above cyclic peptide spectra, the UVPD spectrum requires high mass accuracy and resolving power to assist with the spectral complexity.

Analysis of a poly­(2-ethyl-2-oxazoline) conjugated cyclic peptide by nESI showed four major distributions of ions of two polymeric species, as shown in Figure 3A. The two species present were the cyclic peptide–polymer conjugate and unreacted poly­(2-ethyl-2-oxazoline). The cyclic peptide–polymer conjugate had detectable +4 and +5 protonated ion distributions. The hydrogen-terminated poly­(2-ethyl-2-oxazoline) by product was also protonated with +4 and +5 charge states.

**Figure 3:**
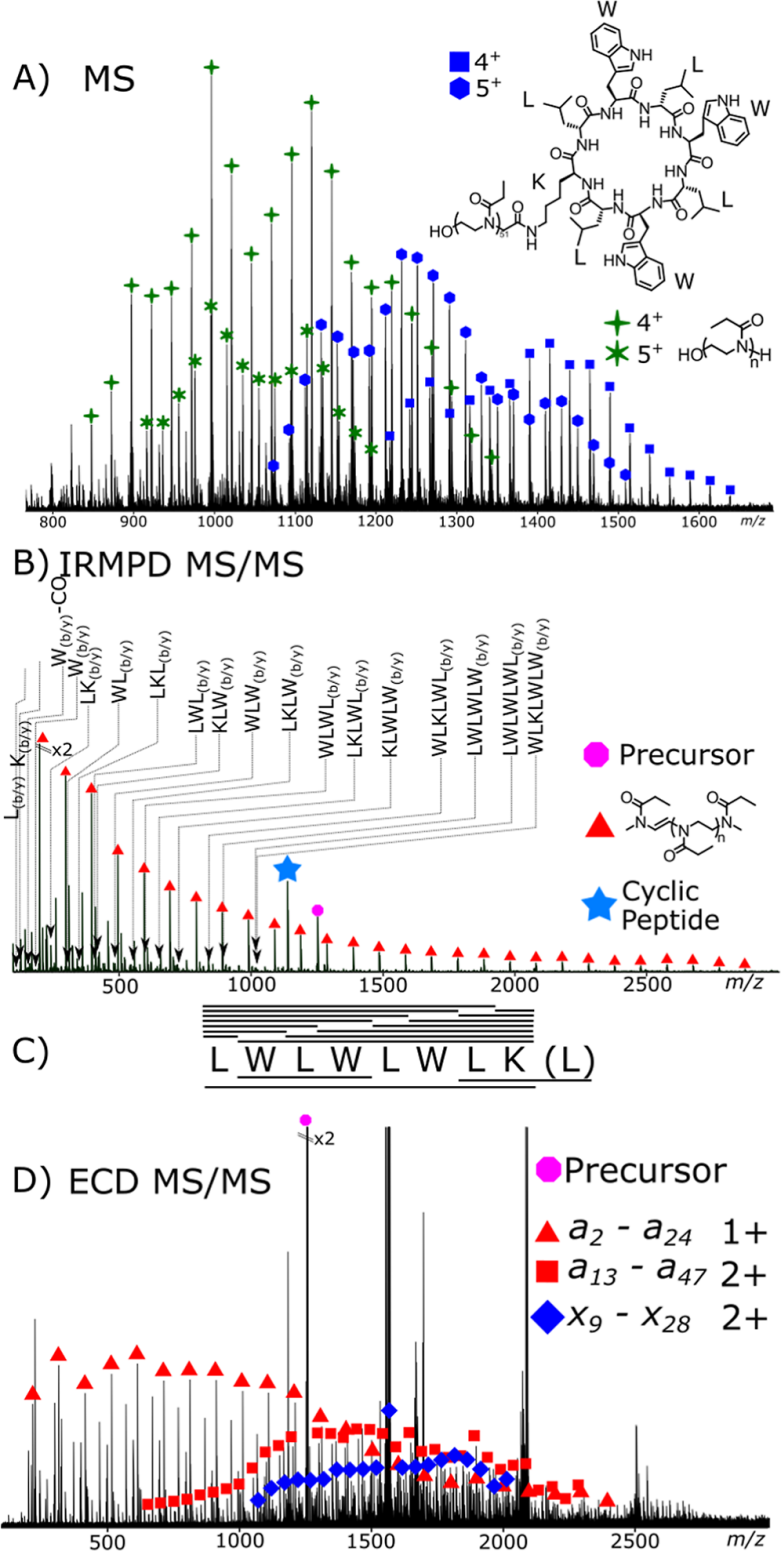
(A) nESI analysis of the cyclic peptide–polymer conjugate showing the presence of +4 and +5 protonated species. The presence of the cyclic peptide–polymer conjugate species and the hydrogen terminated poly­(oxazoline) byproduct is assigned. Tandem mass spectrum of a cyclic peptide polymer conjugate by (B) IRMPD, presents significant cyclic peptide coverage but internal fragmentation of the polymer, 210 summed spectrum scans (C) cyclic peptide coverage and (D) ECD coverage of *a*, OH terminus containing polymer fragments, and *x*, cyclic peptide containing fragments, 100 summed spectrum scans. The +5 charge 51 repeat monomer *m*/*z* 1250.8 was selected for fragmentation for both techniques.

The assigned monomer distribution of the polymer portion of the cyclic peptide–polymer conjugate observed was from 39 monomer units (*m*/*z* 1266.09) to 65 monomer units (*m*/*z*. 1528.23). Monomer assignments for the hydrogen-terminated byproduct were from 30 monomer units to 63 monomer units.

IRMPD fragmentation, Figure 3B, of the cyclic peptide–polymer conjugate showed significant fragmentation of the cyclic peptide, resulting in complete coverage of the cyclic peptide species. Figure 3C presents the fragmentation map of the cyclic peptide with a near-identical fragmentation coverage of the unconjugated cyclic peptide (Figure 2A). The intact cyclic peptide was also detected at a high intensity. The high intensity of the loss of the conjugating polymer can be qualified as being favored over internal cyclic peptide fragmentation due to it being a single fragmentation event of the conjugating amide bond.

The conjugated polymer, though, remained completely uncharacterized by IRMPD MS/MS. Polymer fragment ions consist of rearranged fragments due to the lack of an amide bond in the backbone of the polymer itself. The polymer fragments observed did not contain the terminal fragments and, therefore, did not provide useful sequence information.

ECD fragmentation, Figure 3D, produces a much greater fragment coverage of the conjugating polymer. The *a* series fragments, which contain the terminating OH group span over 4500 Da starting with an *a*_2_ fragment, (singly charged, *m*/*z* 217.15, 0.0 ppm) to an *a*_46_ fragment (doubly charged, *m*/*z* 2288.59, 0.8 ppm). Through the two charge states of fragments, almost complete sequence coverage is observed (94%) in the poly­(oxazoline) polymer. The *z* fragment series, which contains the cyclic peptide conjugated to the polymer is present from *x*_9_ (doubly charged, *m*/*z* 1072.67, −0.27 ppm) to *x*_27_ (doubly charged, *m*/*z* 1964.29, 0.0 ppm). A low intensity singly charged fragment series was observed from *x*_2_ to *x*_11_ (*m*/*z* 1351.79, −0.9 ppm, *m*/*z* 2342.48, 0.3 ppm, respectively). The intensity of the singly charged *z* series was very low and often overlapped by the charge-reduced and neutral loss fragment peaks. The intense peaks observed at higher masses were charge-reduced precursor peaks. The ECD spectrum, unlike the IRMPD spectrum, did not contain any analytically useful cyclic peptide fragments.

Together, IRMPD and ECD offered complementary fragmentation that resulted in a high level of characterization of the cyclic peptide and the cyclic peptide–polymer conjugate but across two experimental analyses.

The analysis of the cyclic peptide–polymer conjugate species by UVPD showed that coverage of the cyclic peptide and the polymer could be achieved within one fragmentation experiment (Figure 4A). UVPD of the cyclic peptide–polymer conjugate produced a rich fragmentation spectrum. The cyclic peptide–polymer conjugate spectrum shows *b*/*y* fragmentation being the major fragmentation pathway for UVPD and IRMPD methods again, consistent with the unconjugated peptide spectra, above. The non-*b*/*y* fragmentation pathways are seen at lower levels (<10% relative intensity) and, with the added complexity of the polymer fragmentation, often drop below detectable levels, although some fragments are present enough to be detected to show that cyclic peptide fragmentation pathways have not changed significantly with the addition of the polymer.

**Figure 4:**
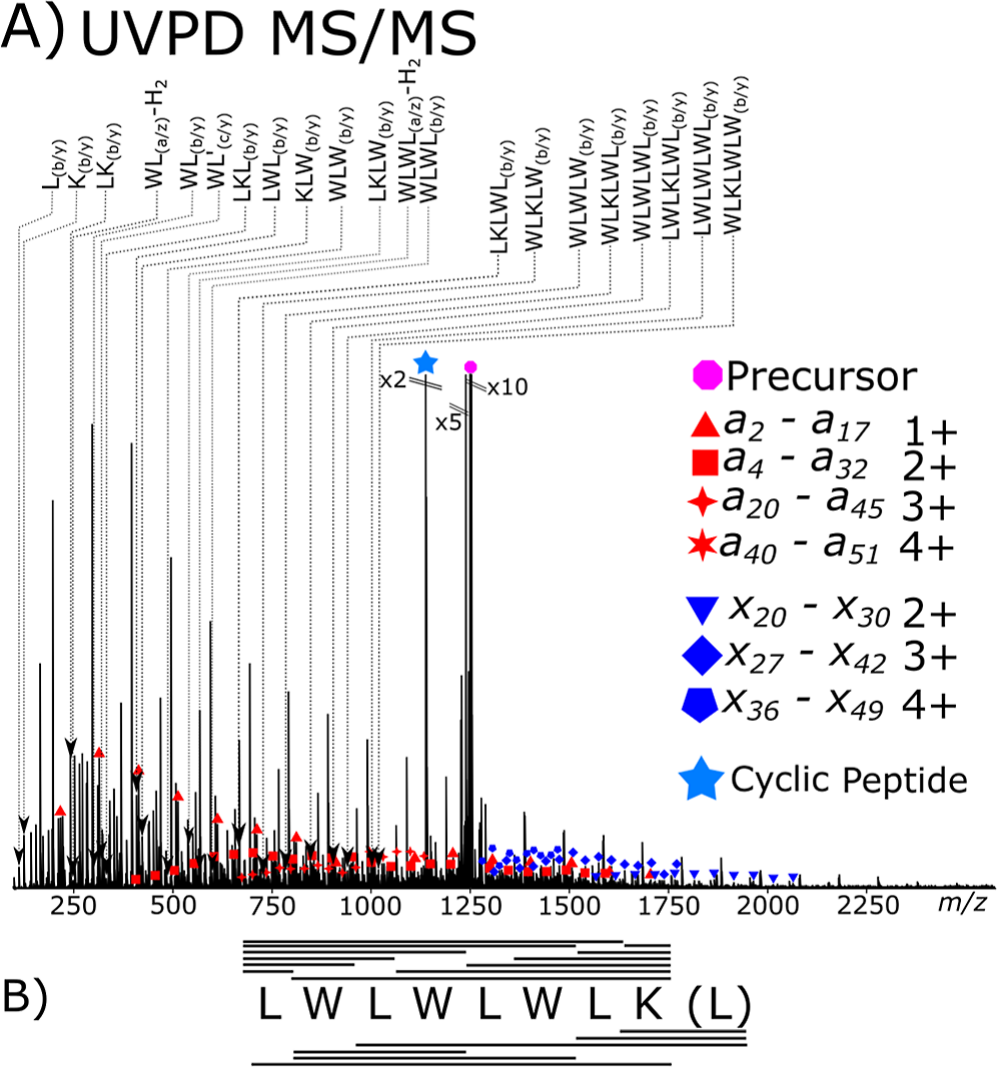
(A) Tandem mass spectrum of a cyclic peptide–polymer conjugate by UVPD showing the *a* and *x* series fragments as well as coverage of the cyclic peptide conjugate species in one experiment, 300 summed spectrum scans. The +5 charge 51 repeat monomer *m*/*z* 1250.8 was selected for fragmentation. (B) Coverage of core cyclic peptide coverage via UVPD fragmentation within the cyclic peptide polymer conjugate.

Sequence *a* and *x* fragments were present, covering the entire polymer backbone sequence, with the *a* fragment series from *a*_2_ (*m*/*z* 217.15, 0.1 ppm) to *a*_51_ (*m*/*z* 1286.63, 0.4 ppm) and the *x* fragments from *x*_20_ (*m*/*z* 1568.02, 0.4 ppm) to *x*_49_ (*m*/*z* 1495.00, −0.2 ppm). Due to the lack of charge reduction in UVPD compared to ECD, the UVPD fragments maintain much broader charge state distribution with large overlaps between fragment series.

The central cyclic peptide was completely sequenced with 22 cyclic peptide-containing fragments, producing 100% cleavage coverage (Figure 4B). The extent to which cyclic peptide scrambling occurs seemed unaffected by the presence of the poly­(2-oxazoline). UVPD fragmentation also produced very similar neutral loss profiles, with many fragments exhibiting water loss (not labeled on the spectrum for clarity; discrete assignments are available in the Supporting Information).

## Conclusions

UVPD MS/MS was shown to be effective in the fragmentation of poly­(2-ethyl-2-oxazoline) polymers producing complete sequence coverage of terminus-containing fragments within complex cyclic-peptide–polymer conjugates.

IRMPD and UVPD produced a complete cleavage coverage of the cyclic peptide species. Although low-intensity sequence scrambling was observed, the intensity of the scrambled fragments was much lower than that of unperturbed sequence fragments. ECD was ineffective at analyzing the cyclic peptide species herein, with observed fragments being the product of side chain losses or sequence scrambled peptide fragments.

The analysis of the cyclic peptide–polymer conjugate could be achieved effectively with the use of IRMPD and ECD MS/MS techniques providing complementary fragmentation to one another, allowing sequencing of the peptide using IRMPD to then be followed by sequencing of the polymer using ECD. UVPD MS/MS allowed both the cyclic peptide and the polymer to be sequenced effectively and extensively in one experiment.

The data present UVPD MS/MS as a robust technique for the analysis of complex polymer-conjugate species, which provides a very useful tool for analytical scientists and characterization of such promising delivery vectors.

## Supplementary Material



## Notes

Supporting information, including mass assignments and NMR and GPC information (PDF)
