# Supplementary material for: Cyclic Peptide–Polymer Conjugate Characterization Using 193 nm Ultraviolet Photodissociation Tandem Mass Spectrometry
Source: Anal Chem. 2026 Feb 5;98(6):4499–507. doi: 10.1021/acs.analchem.5c03375 (PMC12921662; doi:10.1021/acs.analchem.5c03375)
Supplement: Supplementary file 1 [file ac5c03375_si_001.pdf]

SUPPORTING INFORMATION

to

Cyclic peptide-polymer conjugate characterization using 193 nm ultraviolet photodissociation tandem mass spectrometry

by

Tomos E. Morgan[1], Alina Theisen[1], Sean Ellacott[1], Anisha Haris[1], Christopher A. Wootton[1], Julia Y. Rho[1], Mark P. Barrow[1], Anthony W. T. Bristow[2], Sébastien Perrier[1,3,4], Peter B. O'Connor[1*]

[1]Department of Chemistry, University of Warwick, Coventry, CV4 7AL, UK.

[2]Chemical Development, Pharmaceutical Technology & Development, Operations, AstraZeneca, Macclesfield, Cheshire, SK10 2NA

[3]Warwick Medical School, University of Warwick, Coventry, CV4 7AL, UK

[4]Faculty of Pharmacy and Pharmaceutical Sciences, Monash University, 381 Royal Parade, Parkville, VIC 3052, Australia

* Peter B. O'Connor, University of Warwick, UK. Phone: +44 (0)24 76151008; fax: +44 (0)24 76151009;
Email: p.oconnor@warwick.ac.uk;

Contents

Section S1: IRMPD and UVPD implementation on the FT-ICR 2

Section S2: Synthesis of Poly(oxazoline) and species 2

Section S2: Assignment tables 4

Table S 1: UVPD fragment assignment of p(Ox)$S_2OC_3H_5$ homopolymer Figure 1A 5

Table S 2: Internal fragment series from from p(Ox)$S_2OC_3H_5$ 7

Table S 3: Fragments of cyclic peptide: IRMPD Figure 2A 8

Table S 4: Fragments of cyclic peptide: ECD Figure 2C – CRS- charged reduced species 9

Table S 5: Fragments of cyclic peptide: UVPD Figure 2E 10

Table S 6: Intact analysis of cyclic peptide polymer conjugate Figure 3A 11

Table S 7: Cyclic peptide polymer conjugate IRMPD Figure 3B 14

Table S 8: Cyclic peptide polymer conjugate ECD Figure 3D 16

Table S 9: Cyclic peptide polymer conjugate UVPD Figure 4A 18

Section S1: IRMPD and UVPD implementation on the FT-ICR

This section is a summary of the supplementary information from the publication: "Enhancing biomolecule analysis and 2DMS experiments by implementation of (activated ion-) 193nm UVPD on a FT-ICR mass spectrometer"

A commercial 12 T Bruker solariX (Bruker, Bremen, Germany) FT-ICR mass spectrometry equipped with an infinity cell with ECD and IRMPD capabilities was used in this study. The IR laser was triggered by pin J481 on the solariX NICE electronics as installed by Bruker and are unmodified. Pin 35 on the AUX interface J31 at the front of the instrument was chosen as the trigger out for the UV laser. The trigger first goes to an NI PCI card driven by a lab-house LabView program written in-house. The NI card allows the control of the fragmentation parameters of the UV laser, specifically the number of shots, the repetition rate, and the timing of the UV shots after the solariX trigger is received. [1]

The UVPD laser fragmentation occurs while the ions are within the ICR cell before the excitation pulse for detection. In terms of pulse sequence, the IRMPD and UVPD pulse sequences are very similar with fragmentation occurring within a pause between the trapping and the final excitation pulse after ejection of the ions from the flight tube into the ICR cell.

For MS2 data analysis, spectral averaging was implemented: All cyclic peptide spectra were taken with the same number of spectra – 50 spectra. For the Cyclic-peptide polymer conjugate -ECD - 100 scans , IRMPD – 210 scans, UVPD -300 scans

Section S2: Synthesis of Poly(oxazoline) and species

Synthesis of poly(2-ethyl-2-oxazoline): 2-ethyl-2-oxazoline (99%, Sigma Aldrich, Dorset, United Kingdom EtOx) was dried over barium oxide and distilled under reduced pressure then kept in a Schlenk flask prior to use. Methyl p-toluenesulfonate (98%, VWR International Ltd., Lutterworth, United Kingdom), MeTos was distilled under reduced pressure and kept under a nitrogen atmosphere. Potassium ethyl xanthogenae (96%, Sigma Aldrich, Dorset, United Kingdom, potassium ethyl xanthate) Acetonitrile Extra Dry (99.9%+, Fisher Scientific, Acros Organics, Loughborough, United Kingdom)), tert-Butyl bromoacetate (98%, Sigma Aldrich, Dorset, United Kingdom), were used as purchased. 1H NMR spectra were measured using a Bruker DPX-400 NMR spectrometer which operated at 400.05 MHz.

Size exclusion chromatography (SEC) measurements in chloroform (CHCl3) were performed using an Agilent 390-LC MDS (Agilent Technologies LDA UK, Cheadle, United Kingdom) with differential refractive index (DRI), viscometry (VS), dual-angle light scatter (LS) and two wavelength UV detectors. The system was equipped with 2 x PLgel Mixed D columns (300 x 7.5 mm, linear operating range between 200 and 400,000 g mol-1) and a PLgel 5 µm guard column (Agilent Technologies LDA UK, Cheadle, United Kingdom). The eluent was CHCl3 with 2% triethylamine additive. SEC used Polystyrene standards (Agilent Easy Vials (Agilent Technologies LDA UK, Cheadle, United Kingdom) were used for calibration (150-350,000 g mol-1).

Methyl tosylate initiated and xanthate terminated poly(2-ethyl-2-oxazoline)

Polymerisations of 2-ethyl-2-oxazoline were carried on a Biotage Initiator+ microwave synthesizer (Biotage, Uppsala, Sweden). Dry methyl tosylate (0.186 g, 1 mmol), dry 2-ethyl-2-oxazoline (1.983 g, 20 mmol) and extra dry acetonitrile (2.83 mL) were added to a pre-dried Biotage microwave vial, under a constant flux of nitrogen. The vial was sealed, left to stir for 30 s before being heated at 140 °C for 3 min. After cooling, a 2 mL solution of potassium ethyl xanthate (0.192 g, 1.2 mmol) in extra dry acetonitrile was added with a syringe to the polymer mixture for end-capping of the polymer. The solution was left stirring at room temperature for 48 h. Chloroform (50 mL) was and the organic phase was washed three times with a saturated solution of sodium hydrogen carbonate, with brine and dried on magnesium sulfate. The polymer was reconstituted in 10 mL of dichloromethane before precipitation in diethyl ether and dried overnight in a vacuum oven at 40 °C (yield 1.44 g). 1H NMR (400 MHz, CDCl3) δ ppm: 3.75 – 3.13 (m, 80 H, backbone), 3.10 - 2.92 (m, 3 H, Methyl group (α-end)), 2.54 - 2.13 (m, 40 H, CH2 side chain), 1.44 (t, 2.4 H, Methyl group (xanthate)), 1.23 – 0.98 (m, 60 H, CH3 side chain). SEC (CHCl3, trimethylamine, PS calibration): Mn = 2,700 g mol-1, Đ = 1.13.

Synthesis of poly(2-etyl-2-oxazoline) (PEt) for conjugation (terminal hydroxy, carboxy functional)

tert-Butyl bromoacetate (0.062 g, 0.32 mmol), dry 2-ethyl-2-oxazoline (1.42 g, 14.4 mmol) and extra dry acetonitrile (2.50 mL) were transferred to a pre-dried Biotage® microwave vial, under a constant flux of dry nitrogen. The vial was sealed and immediately introduced in the microwave reactor, stirred for 30 s then heated at 140 °C for 50 min. The vial was subsequently unsealed and 30 mL of chloroform were added. The organic phase was washed three times with a saturated solution of sodium hydrogen carbonate, followed by brine and eventually dried on magnesium sulfate. The solvent was removed under reduced pressure and the polymer was redissolved in 10 mL of chloroform before being precipitated three times in diethyl ether. The precipitated polymer was left to dry overnight in a vacuum oven at 40 °C. 500 mg of the formed polymer were then dissolved in 11 ml of a 0.1 M solution of sodium hydroxide and left to stir for 16 h at room temperature. The mixture was treated with Amberlite™ IR120 resin for 30 min before being dialysed once in a 3 kDa centrifuge tube. The polymer solution was then left to freeze-dry, to afford 370 mg of product (white powder). 1H NMR (400 MHz, $CDCl_3$) δ ppm: 3.75 – 3.35 (m, 220 H, backbone), 2.55 – 2.13 (m, 110 H, CH_2 side chain), 1.23 – 0.98 (m, 165 H, CH_3 side chain). SEC (CHCl3, trimethylamine, PS calibration): Mn = 4,500 g mol^{-1}, Đ = 1.28.

Conjugation to 1 arm CP

PEtOx was conjugated to the CP through the carboxyl end group. Here, 23 mg (1 eq., 0,020 mmol) CP was dissolved in DMF. Additionally, 103 mg (1.15 eq., 0,023 mmol) polymer was dissolved in DMF, alongside 8.3 mg HCTU (1 eq., 0,020 mmol), and 6.6 µL NMM (3 eq., 0,060 mmol). The reagents were added together in the order of HCTU/NMM into polymer solution, then polymer solution into CP. The reaction was left for 48 hours to go to completion, as monitored by SEC.

Following conjugation, excess DMF was removed by air drying, and excess water added to the remaining solution. All samples were then purified via centrifuge dialysis, using centrifuge filter tubes (MWCO = 30 kDa, Amicon, Ultracell, UK). Samples were dialysed for a minimum of 3 water changes, followed by lyophilization affording 100 mg of white powder. Purity was checked by SEC analysis.

Size exclusion chromatography (SEC) was carried out on a Polymer Laboratories PL-GPC 50 Plus system using a PolarGel-M guard column (7.5 × 50 mm) followed by two PolarGel-M columns (7.5 × 300 mm). DMF (0.1% LiBr) was used as eluent at 1.0 mL min−1 at 50 °C. Commercial narrow linear poly(methyl

methacrylate) standards in range of 2.0 × 102–1.0 × 106 g mol^{-1} were used to calibrate the DMF SEC system. Analyte samples were filtered through a nylon membrane with 0.22 µm before analysis.

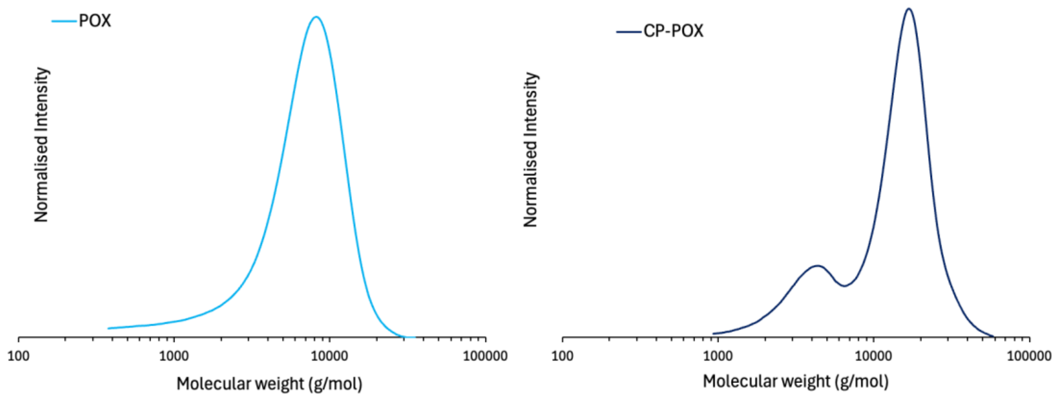

Figure S1. SEC chromatograms for POX polymer and CP-POX conjugate. Eluent in all cases is DMF with 0.1% w/v LiBr. Molecular weights were calculated against a PMMA standard.

Note. The polymer before conjugation shows a unimodal peak (POX, M_n = 3,100 g mol^{-1}, $Đ$ = 1.25). However, unsurprisingly, the CP-POX conjugate shows a bimodal distribution, which can be associated with CP-POX unimer (low molecular weight distribution) and CP-POX dimer (roughly double the molecular weight of the single polymer) due to a small degree of self-assembly. The peak maximum is 8,460 g mol^{-1} (POX) and 17,600 g mol^{-1} (CP-POX), respectively.

Further characterization of CP-POX conjugates can also be found in Biomacromolecules 2021, 22, 2, 710–722.

Characterisation of xanthate terminated poly(2-ethyl-2-oxazoline): Sample used in Figure 1A. Full characterisation presented in publication: Coupling Electron Capture Dissociation and the Modified Kendrick Mass Defect for Sequencing of a Poly(2-ethyl-2-oxazoline) Polymer, Anal. Chem. 2018, 10, 11710.

Section S3: Assignment tables

Internal fragment series structures are given but have not been structurally confirmed by further experiments and offer an example structure that may align with the given chemical formula.

Table S 1: UVPD fragment assignment of p(Ox)$S_2OC_3H_5$ homopolymer Figure 1A

m/z	charge	chemical formula	Error (ppm)	assignment
1078.76049	1	$C_{54}H_{99}N_{11}O_{11}S_0H^+{}_1$	0.61	a_{11}
979.69156	1	$C_{49}H_{90}N_{10}O_{10}S_0H^+{}_1$	0.15	a_{10}
880.62326	1	$C_{44}H_{81}N_9O_9S_0H^+{}_1$	0.29	a_9
781.55461	1	$C_{39}H_{72}N_8O_8S_0H^+{}_1$	0.03	a_8
682.48611	1	$C_{34}H_{63}N_7O_7S_0H^+{}_1$	-0.09	a_7
583.41784	1	$C_{29}H_{54}N_6O_6S_0H^+{}_1$	0.14	a_6
484.34938	1	$C_{24}H_{45}N_5O_5S_0H^+{}_1$	0.07	a_5
385.28101	1	$C_{19}H_{36}N_4O_4S_0H^+{}_1$	0.20	a_4
286.21258	1	$C_{14}H_{27}N_3O_3S_0H^+{}_1$	0.22	a_3
187.14414	1	$C_9H_{18}N_2O_2S_0H^+{}_1$	0.19	a_2
936.1573	2	$C_{94}H_{171}N_{19}O_{19}S_0H^+{}_2$	0.10	a_{19}
886.62555	2	$C_{89}H_{162}N_{18}O_{18}S_0H^+{}_2$	2.87	a_{18}
837.08859	2	$C_{84}H_{153}N_{17}O_{17}S_0H^+{}_2$	-0.24	a_{17}
787.55481	2	$C_{79}H_{144}N_{16}O_{16}S_0H^+{}_2$	0.28	a_{16}
738.02059	2	$C_{74}H_{135}N_{15}O_{15}S_0H^+{}_2$	0.28	a_{15}
688.48632	2	$C_{69}H_{126}N_{14}O_{14}S_0H^+{}_2$	0.21	a_{14}
638.95204	2	$C_{64}H_{117}N_{13}O_{13}S_0H^+{}_2$	0.11	a_{13}
589.41778	2	$C_{59}H_{108}N_{12}O_{12}S_0H^+{}_2$	0.03	a_{12}
539.88369	2	$C_{54}H_{99}N_{11}O_{11}S_0H^+{}_2$	0.25	a_{11}
490.34945	2	$C_{49}H_{90}N_{10}O_{10}S_0H^+{}_2$	0.21	a_{10}
440.81529	2	$C_{44}H_{81}N_9O_9S_0H^+{}_2$	0.34	a_9
624.44061	3	$C_{94}H_{171}N_{19}O_{19}S_0H^+{}_3$	0.07	a_{18}
1212.74621	1	$C_{58}H_{105}N_{11}O_{12}S_2H^+{}_1$	0.31	x_{11}
1113.67801	1	$C_{53}H_{96}N_{10}O_{11}S_2H^+{}_1$	0.53	x_{10}
1014.6093	1	$C_{48}H_{87}N_9O_{10}S_2H^+{}_1$	0.29	x_9
915.54081	1	$C_{43}H_{78}N_8O_9S_2H^+{}_1$	0.24	x_8
816.47195	1	$C_{38}H_{69}N_7O_8S_2H^+{}_1$	-0.28	x_7
717.40376	1	$C_{33}H_{60}N_6O_7S_2H^+{}_1$	-0.01	x_6
618.33554	1	$C_{28}H_{51}N_5O_6S_2H^+{}_1$	0.30	x_5
519.26702	1	$C_{23}H_{42}N_4O_5S_2H^+{}_1$	0.16	x_4
420.19866	1	$C_{18}H_{33}N_3O_4S_2H^+{}_1$	0.32	x_3
321.1302	1	$C_{13}H_{24}N_2O_3S_2H^+{}_1$	0.28	x_2
953.61609	2	$C_{93}H_{168}N_{18}O_{19}S_2H^+{}_2$	0.09	x_{18}
904.08239	2	$C_{88}H_{159}N_{17}O_{18}S_2H^+{}_2$	0.65	x_{17}
854.54798	2	$C_{83}H_{150}N_{16}O_{17}S_2H^+{}_2$	0.46	x_{16}
805.01381	2	$C_{78}H_{141}N_{15}O_{16}S_2H^+{}_2$	0.53	x_{15}
755.47938	2	$C_{73}H_{132}N_{14}O_{15}S_2H^+{}_2$	0.27	x_{14}
656.41073	2	$C_{63}H_{114}N_{12}O_{13}S_2H^+{}_2$	-0.05	x_{12}
606.87663	2	$C_{58}H_{105}N_{11}O_{12}S_2H^+{}_2$	0.12	x_{11}
557.34243	2	$C_{53}H_{96}N_{10}O_{11}S_2H^+{}_2$	0.14	x_{10}
507.80831	2	$C_{48}H_{87}N_9O_{10}S_2H^+{}_2$	0.33	x_9
702.1253	3	$C_{103}H_{186}N_{20}O_{21}S_2H^+{}_3$	-0.10	x_{20}
669.10259	3	$C_{98}H_{177}N_{19}O_{20}S_2H^+{}_3$	0.03	x_{19}
636.07973	3	$C_{93}H_{168}N_{18}O_{19}S_2H^+{}_3$	-0.05	x_{18}
638.4598	1	$C_{32}H_{59}N_7O_6S_0H^+{}_1$	-0.25	1-Internal

539.39162	1	$C_{27}H_{50}N_6O_5S_0H^+_1$	0.14	1-Internal
440.3232	1	$C_{22}H_{41}N_5O_4S_0H^+_1$	0.16	1-Internal
341.25478	1	$C_{17}H_{32}N_4O_3S_0H^+_1$	0.18	1-Internal
242.18639	1	$C_{12}H_{23}N_3O_2S_0H^+_1$	0.36	1-Internal
459.8231	2	$C_{47}H_{83}N_9O_9S_0H^+_2$	0.30	3-Internal
410.28893	2	$C_{42}H_{74}N_8O_8S_0H^+_2$	0.42	3-Internal
360.75473	2	$C_{37}H_{65}N_7O_7S_0H^+_2$	0.50	3-Internal
311.22049	2	$C_{32}H_{56}N_6O_6S_0H^+_2$	0.47	3-Internal
261.68615	2	$C_{27}H_{47}N_5O_5S_0H^+_2$	0.05	3-Internal
212.15203	2	$C_{22}H_{38}N_4O_4S_0H^+_2$	0.47	3-Internal
664.47569	2	$C_{68}H_{122}N_{14}O_{12}S_0H^+_2$	0.12	2-Internal
565.40725	2	$C_{58}H_{104}N_{12}O_{10}S_0H^+_2$	0.10	2-Internal
515.87302	2	$C_{53}H_{95}N_{11}O_9S_0H^+_2$	0.06	2-Internal
466.33891	2	$C_{48}H_{86}N_{10}O_8S_0H^+_2$	0.28	2-Internal
416.80462	2	$C_{43}H_{77}N_9O_7S_0H^+_2$	0.11	2-Internal
367.27045	2	$C_{38}H_{68}N_8O_6S_0H^+_2$	0.22	2-Internal
317.73624	2	$C_{33}H_{59}N_7O_5S_0H^+_2$	0.25	2-Internal
268.20206	2	$C_{28}H_{50}N_6O_4S_0H^+_2$	0.40	2-Internal
882.0691	2	$C_{86}H_{155}N_{17}O_{17}S_2H^+_2$	0.46	4-Internal
832.53467	2	$C_{81}H_{146}N_{16}O_{16}S_2H^+_2$	0.22	4-Internal
783.00072	2	$C_{76}H_{137}N_{15}O_{15}S_2H^+_2$	0.57	4-Internal
733.46622	2	$C_{71}H_{128}N_{14}O_{14}S_2H^+_2$	0.20	4-Internal
683.93217	2	$C_{66}H_{119}N_{13}O_{13}S_2H^+_2$	0.45	4-Internal
634.39789	2	$C_{61}H_{110}N_{12}O_{12}S_2H^+_2$	0.37	4-Internal
584.86349	2	$C_{56}H_{101}N_{11}O_{11}S_2H^+_2$	0.07	4-Internal
535.32931	2	$C_{51}H_{92}N_{10}O_{10}S_2H^+_2$	0.13	4-Internal
485.7952	2	$C_{46}H_{83}N_9O_9S_2H^+_2$	0.34	4-Internal
815.07553	2	$C_{82}H_{149}N_{17}O_{16}S_0H^+_2$	-0.19	5-Internal
765.54134	2	$C_{77}H_{140}N_{16}O_{15}S_0H^+_2$	-0.18	5-Internal
716.00734	2	$C_{72}H_{131}N_{15}O_{14}S_0H^+_2$	0.09	5-Internal
616.93889	2	$C_{62}H_{113}N_{13}O_{12}S_0H^+_2$	0.05	5-Internal
567.4047	2	$C_{57}H_{104}N_{12}O_{11}S_0H^+_2$	0.08	5-Internal
517.87062	2	$C_{52}H_{95}N_{11}O_{10}S_0H^+_2$	0.34	5-Internal
468.33639	2	$C_{47}H_{86}N_{10}O_9S_0H^+_2$	0.32	5-Internal
418.80214	2	$C_{42}H_{77}N_9O_8S_0H^+_2$	0.26	5-Internal
369.26792	2	$C_{37}H_{68}N_8O_7S_0H^+_2$	0.26	5-Internal
510.36502	1	$C_{26}H_{47}N_5O_5S_0H^+_1$	0.05	6-Internal
411.29656	1	$C_{21}H_{38}N_4O_4S_0H^+_1$	-0.05	6-Internal
312.22828	1	$C_{16}H_{29}N_3O_3S_0H^+_1$	0.36	6-Internal
213.15985	1	$C_{11}H_{20}N_2O_2S_0H^+_1$	0.45	6-Internal
793.55463	1	$C_{40}H_{72}N_8O_8S_0H^+_1$	0.05	7-Internal
595.41759	1	$C_{30}H_{54}N_6O_6S_0H^+_1$	-0.29	7-Internal
496.34933	1	$C_{25}H_{45}N_5O_5S_0H^+_1$	-0.03	7-Internal
397.28104	1	$C_{20}H_{36}N_4O_4S_0H^+_1$	0.27	7-Internal
446.21429	1	$C_{20}H_{35}N_3O_4S_2H^+_1$	0.26	7-Internal
347.14586	1	$C_{15}H_{26}N_2O_3S_2H^+_1$	0.28	7-Internal
248.07739	1	$C_{10}H_{17}N_1O_2S_2H^+_1$	0.17	7-Internal

701.79026	3	$C_{103}H_{185}N_{20}O_{21}S_2H^+_3$	1.18	Pre-Me
687.78493	3	$C_{101}H_{183}N_{20}O_{20}S_2H^+_3$	-1.42	Pre-C_3H_5O
682.11815	3	$C_{101}H_{182}N_{20}O_{19}S_2H^+_3$	-0.26	Pre-C_3H_5O-OH
677.1283	3	$C_{101}H_{183}N_{20}O_{20}S_1H^+_3$	-0.37	Pre-C_3H_5OS
673.77459	3	$C_{99}H_{179}N_{19}O_{20}S_2H^+_3$	0.21	Pre-C_5H_9NO
668.77487	3	$C_{98}H_{178}N_{20}O_{19}S_2H^+_3$	0.46	Pre-$_2(C_3H_5O)$
666.47129	3	$C_{101}H_{183}N_{20}O_{20}S_0H^+_3$	0.14	Pre-$C_5H_5OS_2$
658.76306	3	$C_{97}H_{174}N_{19}O_{19}S_2H^+_3$	-0.07	Pre-$C_7H_{14}NO_2$
658.11781	3	$C_{98}H_{178}N_{20}O_{19}S_1H^+_3$	0.92	Pre-C_3H_5O-C_3H_5OS
657.43923	3	$C_{98}H_{174}N_{19}O_{20}S_1H^+_3$	2.81	Pre-C_3H_5OS-C_3H_9N
647.45989	3	$C_{98}H_{178}N_{20}O_{19}S_0H^+_3$	0.07	Pre-$C_5H_5OS_2$-C_3H_5O
633.11252	3	$C_{96}H_{173}N_{19}O_{19}S_0H^+_3$	0.11	Pre-$C_8H_{14}NO_2S_2$-H
632.7605	3	$C_{94}H_{166}N_{20}O_{20}S_0H^+_3$	0.50	Pre-$C_5H_9OS_2$-C_5H_{13}
		Average	0.4	
		Standard Deviation	0.67	

Table S 2: Internal fragment series from from p(Ox)$S_2OC_3H_5$

Fragment	Initial fragment mass	Error (ppm)	Ref Code (table above)
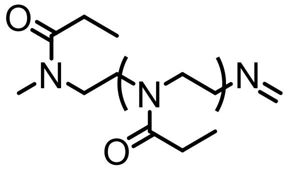	242.186303	0.4	1
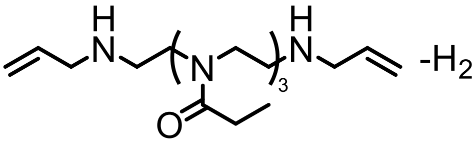	268.201954 (2+)	0.4	2
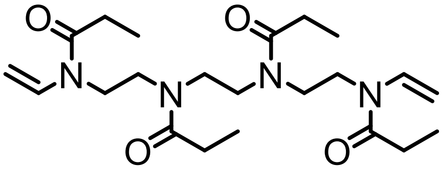	212.151929 (2+)	0.4	3
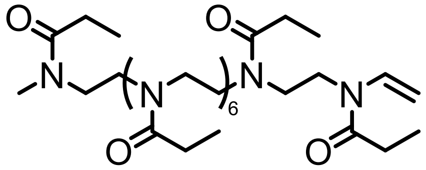	485.795035 (2+)	0.3	4
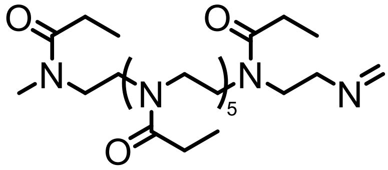	369.267825 (2+)	0.5	5
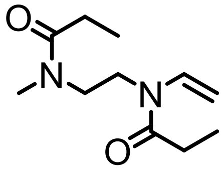	213.15975	0.4	6
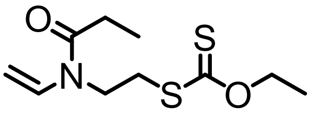	248.077347	0.2	7

Table S 3: Fragments of cyclic peptide: IRMPD Figure 2A

m/z	charge	chemical formula	error	Assignment
129.10227	1	$C_6H_{12}N_2O_1H+_1$	0.24	K (b/y)
242.1863	1	$C_{12}H_{23}N_3O_2H+_1$	-0.01	LK (b/y)
300.17064	1	$C_{17}H_{21}N_3O_2H+_1$	-0.04	WL (b/y)
327.27539	1	$C_{17}H_{34}N_4O_2H+_1$	-0.19	LKL -CO
355.27035	1	$C_{18}H_{34}N_4O_3H+_1$	-0.05	LKL (b/y)
413.2547	1	$C_{23}H_{32}N_4O_3H+_1$	-0.04	LWL (b/y)
428.26561	1	$C_{23}H_{33}N_5O_3H+_1$	-0.01	KLW (b/y)
486.24997	1	$C_{28}H_{31}N_5O_3H+_1$	0.01	WLW (b/y)
541.34966	1	$C_{29}H_{44}N_6O_4H+_1$	-0.04	LKLW (b/y)
599.334	1	$C_{34}H_{42}N_6O_4H+_1$	-0.05	WLWL (b/y)
654.43376	1	$C_{35}H_{55}N_7O_5H+_1$	0.02	LKLWL (b/y)
712.4181	1	$C_{40}H_{53}N_7O_5H+_1$	0.01	LWLWL (b/y)
727.42897	1	$C_{40}H_{54}N_8O_5H+_1$	-0.03	KLWLW (b/y)
785.41348	1	$C_{45}H_{52}N_8O_5H+_1$	0.17	WLWLW (b/y)
840.51292	1	$C_{46}H_{65}N_9O_6H+_1$	-0.16	LKLWLW (b/y)
898.49731	1	$C_{51}H_{63}N_9O_6H+_1$	-0.11	LWLWLW (b/y)
953.59678	1	$C_{52}H_{76}N_{10}O_7H+_1$	-0.36	LKLWLWL (b/y)
1011.58145	1	$C_{57}H_{74}N_{10}O_7H+_1$	-0.02	LWLWLWL (b/y)
1026.59187	1	$C_{57}H_{75}N_{11}O_7H+_1$	-0.49	LWKWLWL (b/y)
224.17574	1	$C_{12}H_{21}N_3O_1H+_1$	0.01	LK (b/y)-H_2O
282.16008	1	$C_{17}H_{19}N_3O_1H+_1$	-0.03	WL (b/y)-H_2O
337.25979	1	$C_{18}H_{32}N_4O_2H+_1$	-0.04	LKL (b/y)-H_2O
395.24415	1	$C_{23}H_{30}N_4O_2H+_1$	-0.01	LWL (b/y)-H_2O
410.25505	1	$C_{23}H_{31}N_5O_2H+_1$	0.00	KLW (b/y)-H_2O
468.23945	1	$C_{28}H_{29}N_5O_2H+_1$	0.10	WLW (b/y)-H_2O
523.33913	1	$C_{29}H_{42}N_6O_3H+_1$	0.03	LKLW (b/y)-H_2O
581.32349	1	$C_{34}H_{40}N_6O_3H+_1$	0.04	WLWL (b/y)-H_2O
636.42319	1	$C_{35}H_{53}N_7O_4H+_1$	0.02	LKLWL (b/y)-H_2O
694.40751	1	$C_{40}H_{51}N_7O_4H+_1$	-0.03	LWLWL (b/y)-H_2O
709.41828	1	$C_{40}H_{52}N_8O_4H+_1$	-0.21	KLWLW (b/y)-H_2O
767.4028	1	$C_{45}H_{50}N_8O_4H+_1$	0.03	WLWLW (b/y)-H_2O
822.50255	1	$C_{46}H_{63}N_9O_5H+_1$	0.07	LKLWLW (b/y)-H_2O
880.48678	1	$C_{51}H_{61}N_9O_5H+_1$	-0.07	LWLWLW (b/y)-H_2O
935.58663	1	$C_{52}H_{74}N_{10}O_6H+_1$	0.08	LKLWLWL (b/y)-H_2O
993.57041	1	$C_{57}H_{72}N_{10}O_6H+_1$	-0.50	LWLWLWL (b/y)-H_2O
197.16484	1	$C_{11}H_{20}N_2O_1H+_1$	0.00	LK (a/z) -H_2
210.12773	1	$C_{15}H_{15}N_1O_0H+_1$	0.02	WL-2(CO-NH_3)
255.14918	1	$C_{16}H_{18}N_2O_1H+_1$	-0.04	WL (a/z) -H_2
310.2489	1	$C_{17}H_{31}N_3O_2H+_1$	-0.01	LKL-(a/z) -H_2
368.23324	1	$C_{22}H_{29}N_3O_2H+_1$	-0.04	LWL (a/z) -H_2
383.24417	1	$C_{22}H_{30}N_4O_2H+_1$	0.05	KLW (a/z)-H_2
441.2285	1	$C_{27}H_{28}N_4O_2H+_1$	-0.01	WLW (a/z)-H_2
496.32833	1	$C_{28}H_{41}N_5O_3H+_1$	0.23	LKLW (a/z) -H_2
554.3126	1	$C_{33}H_{39}N_5O_3H+_1$	0.06	WLWL (a/z)-H_2
667.39661	1	$C_{39}H_{50}N_6O_4H+_1$	-0.03	LWLWL (a/z) -H_2

682.40723	1	$C_{39}H_{51}N_7O_4H+_1$	-0.44	KLWLW (a/z) -H_2
740.39213	1	$C_{44}H_{49}N_7O_4H+_1$	0.34	WLWLW (a/z) -H_2
853.47559	1	$C_{50}H_{60}N_8O_5H+_1$	-0.41	LWLWLW (a/z) -H_2
908.57607	1	$C_{51}H_{73}N_9O_6H+_1$	0.45	LKLWLWL (a/z) -H_2
966.55988	1	$C_{56}H_{71}N_9O_6H+_1$	-0.13	LWLWLWL (a/z) -H_2
227.1754	1	$C_{12}H_{22}N_2O_2H+_1$	-0.02	LL (b/y)
272.17573	1	$C_{16}H_{21}N_3O_1H+_1$	-0.03	KW (a/z) -H_2
373.16589	1	$C_{22}H_{20}N_4O_2H+_1$	-0.03	WW (b/y)
596.33421	1	$C_{34}H_{41}N_7O_3H+_1$	-0.26	WKLW (b/y)-H_2O
614.34494	1	$C_{34}H_{43}N_7O_4H+_1$	0.02	WKLW (b/y)

Table S 4: Fragments of cyclic peptide: ECD Figure 2C – CRS- charged reduced species

**m/z**	**charge**	**chemical formula**	**Error (ppm)**	**Assignment**
428.26561	1	$C_{23}H_{33}N_5O_3H+_1$	-0.01	LWL b/c
541.34969	1	$C_{29}H_{44}N_6O_4H+_1$	0.02	LLWL b/c
570.34154	2	$C_{63}H_{86}N_{12}O_8H+_2$	-0.55	Precursor
727.42897	1	$C_{40}H_{54}N_8O_5H+_1$	-0.03	LLWLW b/c
840.51315	1	$C_{46}H_{65}N_9O_6H+_1$	0.11	LWLLWL b/c
899.50157	1	$C_{46}H_{64}N_{11}O_8H+_1$	0.40	CRS-$C_{17}H_{22}N$
955.56391	1	$C_{50}H_{72}N_{11}O_8H+_1$	0.10	CRS-$C_{13}H_{14}N$
968.5718	1	$C_{51}H_{73}N_{11}O_8H+_1$	0.17	CRS-$C_{12}H_{13}N$
983.58595	1	$C_{56}H_{74}N_{10}O_6H+_1$	-0.62	CRS-$C_7H_{12}N_2O_2$
1011.62657	1	$C_{54}H_{80}N_{11}O_8H+_1$	0.16	CRS-C_9H_6N
1024.6346	1	$C_{55}H_{81}N_{11}O_8H+_1$	0.36	CRS-C_8H_5N
1028.55891	1	$C_{55}H_{71}N_{12}O_8H+_1$	-0.14	CRS-C_8H_{15}
1041.56701	1	$C_{56}H_{72}N_{12}O_8H+_1$	0.12	CRS-C_7H_{14}
1067.5948	1	$C_{59}H_{76}N_{11}O_8H+_1$	-0.29	CRS-$C_4H_{10}N$
1084.62207	1	$C_{59}H_{79}N_{12}O_8H+_1$	0.38	CRS-C_4H_7
1097.62928	1	$C_{60}H_{80}N_{12}O_8H+_1$	-0.19	CRS-C_3H_6
1122.67463	1	$C_{63}H_{85}N_{12}O_7H+_1$	0.83	Precursor-OH
1139.67645	1	$C_{63}H_{86}N_{12}O_8H+_1$	0.01	CRS
			0.05	

Table S 5: Fragments of cyclic peptide: UVPD Figure 2E

m/z	Charge	Chemical formula	Error	Assignment
129.10235	1	$C_6H_{12}N_2O_1H+_1$	0.86	K (b/y)
428.26561	1	$C_{23}H_{33}N_5O_3H+_1$	-0.01	KLW (b/y)
410.25504	1	$C_{23}H_{31}N_5O_2H+_1$	-0.03	KLW (b/y)-H_2O
727.42911	1	$C_{40}H_{54}N_8O_5H+_1$	0.16	KLWLW (b/y)
709.41887	1	$C_{40}H_{52}N_8O_4H+_1$	0.62	KLWLW (b/y)-H_2O
242.1863	1	$C_{12}H_{23}N_3O_2H+_1$	-0.01	LK (b/y)
224.17575	1	$C_{12}H_{21}N_3O_1H+_1$	0.05	LK (b/y)-H_2O
355.27033	1	$C_{18}H_{34}N_4O_3H+_1$	-0.11	LKL (b/y)
337.25974	1	$C_{18}H_{32}N_4O_2H+_1$	-0.19	LKL(b/y)-H_2O
541.34969	1	$C_{29}H_{44}N_6O_4H+_1$	0.02	LKLW (b/y)
523.33918	1	$C_{29}H_{42}N_6O_3H+_1$	0.12	LKLW (b/y)-H_2O
654.43381	1	$C_{35}H_{55}N_7O_5H+_1$	0.10	LKLWL (b/y)
636.42314	1	$C_{35}H_{53}N_7O_4H+_1$	-0.06	LKLWL (b/y)-H_2O
840.51297	1	$C_{46}H_{65}N_9O_6H+_1$	-0.10	LKLWLW (b/y)
822.50246	1	$C_{46}H_{63}N_9O_5H+_1$	-0.04	LKLWLW (b/y) -H_2O
953.59574	1	$C_{52}H_{76}N_{10}O_7H+_1$	-1.45	LKLWLWL (b/y)
413.25468	1	$C_{23}H_{32}N_4O_3H+_1$	-0.09	LWL (b/y)
395.24409	1	$C_{23}H_{30}N_4O_2H+_1$	-0.16	LWL (b/y)-H_2O
712.41811	1	$C_{40}H_{53}N_7O_5H+_1$	0.02	LWLWL (b/y)
694.40773	1	$C_{40}H_{51}N_7O_4H+_1$	0.29	LWLWL (b/y)-H_2O
898.49751	1	$C_{51}H_{63}N_9O_6H+_1$	0.11	LWLWLW (b/y)
880.48682	1	$C_{51}H_{61}N_9O_5H+_1$	-0.03	LWLWLW (b/y)-H_2O
1011.58146	1	$C_{57}H_{74}N_{10}O_7H+_1$	-0.01	LWLWLWL (b/y)
993.57169	1	$C_{57}H_{72}N_{10}O_6H+_1$	0.79	LWLWLWL (b/y)-H_2O
187.08663	1	$C_{11}H_{10}N_2O_1H+_1$	0.22	W (b/y)
169.07608	1	$C_{11}H_8N_2O_0H+_1$	0.33	W(b/y)-H_2O
300.17063	1	$C_{17}H_{21}N_3O_2H+_1$	-0.08	WL (b/y)
282.16006	1	$C_{17}H_{19}N_3O_1H+_1$	-0.10	WL (b/y)-H_2O
486.24996	1	$C_{28}H_{31}N_5O_3H+_1$	-0.01	WLW (b/y)
468.23927	1	$C_{28}H_{29}N_5O_2H+_1$	-0.28	WLW (b/y)-H_2O
599.33403	1	$C_{34}H_{42}N_6O_4H+_1$	0.00	WLWL (b/y)
581.32355	1	$C_{34}H_{40}N_6O_3H+_1$	0.15	WLWL (b/y)-H_2O
785.41381	1	$C_{45}H_{52}N_8O_5H+_1$	0.59	WLWLW (b/y)
767.4028	1	$C_{45}H_{50}N_8O_4H+_1$	0.03	WLWLW (b/y)-H_2O
1026.59092	1	$C_{57}H_{75}N_{11}O_7H+_1$	-1.41	WLWLWLK (b/y)
383.24407	1	$C_{22}H_{30}N_4O_2H+_1$	-0.22	KLW (a/z)-H_2
365.23357	1	$C_{22}H_{28}N_4O_1H+_1$	-0.05	KLW (a/z)-H_2 -H_2O
682.4074	1	$C_{39}H_{51}N_7O_4H+_1$	-0.19	KLWLW (a/z) -H_2
272.17572	1	$C_{16}H_{21}N_3O_1H+_1$	-0.07	KW (a/z) -H_2
254.16515	1	$C_{16}H_{19}N_3O_0H+_1$	-0.09	KW (a/z) -H_2 -H_2O
197.16487	1	$C_{11}H_{20}N_2O_1H+_1$	0.15	LK (a/z) -H_2
227.17541	1	$C_{12}H_{22}N_2O_2H+_1$	0.02	LK (b/z)
310.24881	1	$C_{17}H_{31}N_3O_2H+_1$	-0.30	LKL-(a/z) -H_2
496.32811	1	$C_{28}H_{41}N_5O_3H+_1$	-0.21	LKLW (a/z) -H_2
795.49167	1	$C_{45}H_{62}N_8O_5H+_1$	0.10	LKLWLW (a/z) -H_2
368.23322	1	$C_{22}H_{29}N_3O_2H+_1$	-0.09	LWL (a/z) -H_2
350.22264	1	$C_{22}H_{27}N_3O_1H+_1$	-0.14	LWL (a/z) -H_2 -H_2O

667.39649	1	$C_{39}H_{50}N_6O_4H+_1$	-0.21	LWLWL (a/z) -H_2
966.55575	1	$C_{56}H_{71}N_9O_6H+_1$	-4.40	LWLWLWL (a/z) -H_2
255.14919	1	$C_{16}H_{18}N_2O_1H+_1$	0.00	WL (a/z) -H_2
237.13861	1	$C_{16}H_{16}N_2O_0H+_1$	-0.06	WL (a/z) -H_2 -H_2O
302.18626	1	$C_{17}H_{23}N_3O_2H+_1$	-0.14	WL (c/z) +H
441.2285	1	$C_{27}H_{28}N_4O_2H+_1$	-0.01	WLW (a/z)-H_2
554.31258	1	$C_{33}H_{39}N_5O_3H+_1$	0.02	WLWL (a/z)-H_2
740.39192	1	$C_{44}H_{49}N_7O_4H+_1$	0.05	WLWLW (a/z) -H_2
614.34505	1	$C_{34}H_{43}N_7O_4H+_1$	0.20	WLWL (c/y)
204.11316	1	$C_{11}H_{13}N_3O_1H+_1$	0.11	W (c/y)+H
189.10227	1	$C_{11}H_{12}N_2O_1H+_1$	0.16	W (c/z) +H
159.09174	1	$C_{10}H_{10}N_2O_0H+_1$	0.41	W(a/y)
310.15495	1	$C_{18}H_{19}N_3O_2H+_1$	-0.17	WL (b/x) -H_2O
317.19717	1	$C_{17}H_{24}N_4O_2H+_1$	-0.10	WL (c/y) +H
285.15972	1	$C_{17}H_{20}N_2O_2H+_1$	-0.12	WL(b/z)
445.29208	1	$C_{23}H_{36}N_6O_3H+_1$	-0.19	WLK (c/y) +H
430.28127	1	$C_{23}H_{35}N_5O_3H+_1$	0.01	WLK (c/z) +H
558.37602	1	$C_{29}H_{47}N_7O_4H+_1$	-0.38	WLKL (c/y) +H
543.36536	1	$C_{29}H_{46}N_6O_4H+_1$	0.05	WLKL (c/z) +H
488.26576	1	$C_{28}H_{33}N_5O_3H+_1$	0.29	WLW (c/z) +H
570.34142	2	$C_{63}H_{86}N_{12}O_8H+_2$	-0.76	Precursor
373.1658	1	$C_{22}H_{20}N_4O_2H+_1$	-0.27	WW (b/y)

Table S 6: Intact analysis of cyclic peptide polymer conjugate Figure 3A

m/z	Charge	Chemical Formula	Error (ppm)	Assignment
1216.55888	4	$C_{250}H_{421}N_{49}O_{47}S_0H^+_4$	0.09	(Cycpep)$(Pox)_{38}$ $4H^+$
1266.09387	4	$C_{260}H_{439}N_{51}O_{49}S_0H^+_4$	0.71	(Cycpep)$(Pox)_{39}$ $4H^+$
1290.85677	4	$C_{265}H_{448}N_{52}O_{50}S_0H^+_4$	-2.56	(Cycpep)$(Pox)_{40}$ $4H^+$
1315.61917	4	$C_{270}H_{457}N_{53}O_{51}S_0H^+_4$	-6.09	(Cycpep)$(Pox)_{41}$ $4H^+$
1340.39152	4	$C_{275}H_{466}N_{54}O_{52}S_0H^+_4$	-2.06	(Cycpep)$(Pox)_{42}$ $4H^+$
1365.15289	4	$C_{280}H_{475}N_{55}O_{53}S_0H^+_4$	-6.22	(Cycpep)$(Pox)_{43}$ $4H^+$
1389.92669	4	$C_{285}H_{484}N_{56}O_{54}S_0H^+_4$	-1.29	(Cycpep)$(Pox)_{44}$ $4H^+$
1414.68783	4	$C_{290}H_{493}N_{57}O_{55}S_0H^+_4$	-5.49	(Cycpep)$(Pox)_{45}$ $4H^+$
1439.4529	4	$C_{295}H_{502}N_{58}O_{56}S_0H^+_4$	-6.81	(Cycpep)$(Pox)_{46}$ $4H^+$
1464.22244	4	$C_{300}H_{511}N_{59}O_{57}S_0H^+_4$	-5.03	(Cycpep)$(Pox)_{47}$ $4H^+$
1488.99187	4	$C_{305}H_{520}N_{60}O_{58}S_0H^+_4$	-3.38	(Cycpep)$(Pox)_{48}$ $4H^+$
1513.75891	4	$C_{310}H_{529}N_{61}O_{59}S_0H^+_4$	-3.37	(Cycpep)$(Pox)_{49}$ $4H^+$
1538.52342	4	$C_{315}H_{538}N_{62}O_{60}S_0H^+_4$	-5	(Cycpep)$(Pox)_{50}$ $4H^+$
1563.28744	4	$C_{320}H_{547}N_{63}O_{61}S_0H^+_4$	-6.89	(Cycpep)$(Pox)_{51}$ $4H^+$
1588.05683	4	$C_{325}H_{556}N_{64}O_{62}S_0H^+_4$	-5.34	(Cycpep)$(Pox)_{52}$ $4H^+$
1612.83079	4	$C_{330}H_{565}N_{65}O_{63}S_0H^+_4$	-1.01	(Cycpep)$(Pox)_{53}$ $4H^+$
1637.59674	4	$C_{335}H_{574}N_{66}O_{64}S_0H^+_4$	-1.7	(Cycpep)$(Pox)_{54}$ $4H^+$
1013.07542	5	$C_{260}H_{439}N_{51}O_{49}S_0H^+_5$	-0.41	(Cycpep)$(Pox)_{41}$ $5H^+$
1032.88715	5	$C_{265}H_{448}N_{52}O_{50}S_0H^+_5$	-2.29	(Cycpep)$(Pox)_{42}$ $5H^+$

1072.51415	5	$C_{275}H_{466}N_{54}O_{52}S_0H^+_5$	-2.55	(Cycpep)(Pox)$_{43}$ 5H$^+$
1092.33047	5	$C_{280}H_{475}N_{55}O_{53}S_0H^+_5$	-0.09	(Cycpep)(Pox)$_{44}$ 5H$^+$
1112.14362	5	$C_{285}H_{484}N_{56}O_{54}S_0H^+_5$	-0.56	(Cycpep)(Pox)$_{45}$ 5H$^+$
1131.95845	5	$C_{290}H_{493}N_{57}O_{55}S_0H^+_5$	0.46	(Cycpep)(Pox)$_{46}$ 5H$^+$
1151.76931	5	$C_{295}H_{502}N_{58}O_{56}S_0H^+_5$	-2	(Cycpep)(Pox)$_{47}$ 5H$^+$
1171.58213	5	$C_{300}H_{511}N_{59}O_{57}S_0H^+_5$	-2.7	(Cycpep)(Pox)$_{48}$ 5H$^+$
1191.3958	5	$C_{305}H_{520}N_{60}O_{58}S_0H^+_5$	-2.67	(Cycpep)(Pox)$_{49}$ 5H$^+$
1211.21059	5	$C_{310}H_{529}N_{61}O_{59}S_0H^+_5$	-1.71	(Cycpep)(Pox)$_{50}$ 5H$^+$
1250.83863	5	$C_{320}H_{547}N_{63}O_{61}S_0H^+_5$	-1.12	(Cycpep)(Pox)$_{51}$ 5H$^+$
1270.65083	5	$C_{325}H_{556}N_{64}O_{62}S_0H^+_5$	-2.27	(Cycpep)(Pox)$_{52}$ 5H$^+$
1290.46498	5	$C_{330}H_{565}N_{65}O_{63}S_0H^+_5$	-1.87	(Cycpep)(Pox)$_{53}$ 5H$^+$
1310.27935	5	$C_{335}H_{574}N_{66}O_{64}S_0H^+_5$	-1.32	(Cycpep)(Pox)$_{54}$ 5H$^+$
1330.08618	5	$C_{340}H_{583}N_{67}O_{65}S_0H^+_5$	-6.45	(Cycpep)(Pox)$_{55}$ 5H$^+$
1349.90252	5	$C_{345}H_{592}N_{68}O_{66}S_0H^+_5$	-4.39	(Cycpep)(Pox)$_{56}$ 5H$^+$
1369.7193	5	$C_{350}H_{601}N_{69}O_{67}S_0H^+_5$	-2.06	(Cycpep)(Pox)$_{57}$ 5H$^+$
1389.53289	5	$C_{355}H_{610}N_{70}O_{68}S_0H^+_5$	-2.1	(Cycpep)(Pox)$_{58}$ 5H$^+$
1409.34737	5	$C_{360}H_{619}N_{71}O_{69}S_0H^+_5$	-1.5	(Cycpep)(Pox)$_{59}$ 5H$^+$
1429.16156	5	$C_{365}H_{628}N_{72}O_{70}S_0H^+_5$	-1.13	(Cycpep)(Pox)$_{60}$ 5H$^+$
1448.97571	5	$C_{370}H_{637}N_{73}O_{71}S_0H^+_5$	-0.79	(Cycpep)(Pox)$_{61}$ 5H$^+$
1468.78819	5	$C_{375}H_{646}N_{74}O_{72}S_0H^+_5$	-1.6	(Cycpep)(Pox)$_{62}$ 5H$^+$
1488.59739	5	$C_{380}H_{655}N_{75}O_{73}S_0H^+_5$	-4.59	(Cycpep)(Pox)$_{63}$ 5H$^+$
1508.40781	5	$C_{385}H_{664}N_{76}O_{74}S_0H^+_5$	-6.69	(Cycpep)(Pox)$_{64}$ 5H$^+$
1528.22566	5	$C_{390}H_{673}N_{77}O_{75}S_0H^+_5$	-3.88	(Cycpep)(Pox)$_{65}$ 5H$^+$
748.5238	4	$C_{150}H_{272}N_{30}O_{31}S_0H^+_4$	1.04	H-terminated 4H$^+$
773.29112	4	$C_{155}H_{281}N_{31}O_{32}S_0H^+_4$	1.29	H-terminated 4H$^+$
798.05744	4	$C_{160}H_{290}N_{32}O_{33}S_0H^+_4$	0.26	H-terminated 4H$^+$
822.82433	4	$C_{165}H_{299}N_{33}O_{34}S_0H^+_4$	0	H-terminated 4H$^+$
847.59173	4	$C_{170}H_{308}N_{34}O_{35}S_0H^+_4$	0.35	H-terminated 4H$^+$
872.3579	4	$C_{175}H_{317}N_{35}O_{36}S_0H^+_4$	-0.73	H-terminated 4H$^+$
897.12555	4	$C_{180}H_{326}N_{36}O_{37}S_0H^+_4$	-0.1	H-terminated 4H$^+$
921.89187	4	$C_{185}H_{335}N_{37}O_{38}S_0H^+_4$	-0.95	H-terminated 4H$^+$
946.65981	4	$C_{190}H_{344}N_{38}O_{39}S_0H^+_4$	-0.04	H-terminated 4H$^+$
971.42593	4	$C_{195}H_{353}N_{39}O_{40}S_0H^+_4$	-1.05	H-terminated 4H$^+$
996.19485	4	$C_{200}H_{362}N_{40}O_{41}S_0H^+_4$	0.8	H-terminated 4H$^+$
1020.95943	4	$C_{205}H_{371}N_{41}O_{42}S_0H^+_4$	-1.69	H-terminated 4H$^+$
1045.72626	4	$C_{210}H_{380}N_{42}O_{43}S_0H^+_4$	-1.92	H-terminated 4H$^+$
1070.49555	4	$C_{215}H_{389}N_{43}O_{44}S_0H^+_4$	0.17	H-terminated 4H$^+$
1095.26043	4	$C_{220}H_{398}N_{44}O_{45}S_0H^+_4$	-1.86	H-terminated 4H$^+$
1120.0289	4	$C_{225}H_{407}N_{45}O_{46}S_0H^+_4$	-0.6	H-terminated 4H$^+$
1144.79449	4	$C_{230}H_{416}N_{46}O_{47}S_0H^+_4$	-1.91	H-terminated 4H$^+$
1169.5627	4	$C_{235}H_{425}N_{47}O_{48}S_0H^+_4$	-0.92	H-terminated 4H$^+$
1194.33023	4	$C_{240}H_{434}N_{48}O_{49}S_0H^+_4$	-0.55	H-terminated 4H$^+$
1243.86529	4	$C_{250}H_{452}N_{50}O_{51}S_0H^+_4$	0.16	H-terminated 4H$^+$
1268.63219	4	$C_{255}H_{461}N_{51}O_{52}S_0H^+_4$	0	H-terminated 4H$^+$
1293.39964	4	$C_{260}H_{470}N_{52}O_{53}S_0H^+_4$	0.26	H-terminated 4H$^+$
1318.16041	4	$C_{265}H_{479}N_{53}O_{54}S_0H^+_4$	-4.55	H-terminated 4H$^+$
1342.92703	4	$C_{270}H_{488}N_{54}O_{55}S_0H^+_4$	-4.82	H-terminated 4H$^+$
1367.69421	4	$C_{275}H_{497}N_{55}O_{56}S_0H^+_4$	-4.68	H-terminated 4H$^+$
1392.46358	4	$C_{280}H_{506}N_{56}O_{57}S_0H^+_4$	-2.97	H-terminated 4H$^+$

1417.22599	4	$C_{285}H_{515}N_{57}O_{58}S_{0}H^{+}_{4}$	-6.23	H-terminated 4H⁺
1441.99608	4	$C_{290}H_{524}N_{58}O_{59}S_{0}H^{+}_{4}$	-4.05	H-terminated 4H⁺
700.48991	3	$C_{105}H_{191}N_{21}O_{22}S_{0}H^{+}_{3}$	0.31	H-terminated 3H⁺
733.5136	3	$C_{110}H_{200}N_{22}O_{23}S_{0}H^{+}_{3}$	1.5	H-terminated 3H⁺
766.53582	3	$C_{115}H_{209}N_{23}O_{24}S_{0}H^{+}_{3}$	0.67	H-terminated 3H⁺
799.559	3	$C_{120}H_{218}N_{24}O_{25}S_{0}H^{+}_{3}$	1.11	H-terminated 3H⁺
832.5815	3	$C_{125}H_{227}N_{25}O_{26}S_{0}H^{+}_{3}$	0.7	H-terminated 3H⁺
865.60341	3	$C_{130}H_{236}N_{26}O_{27}S_{0}H^{+}_{3}$	-0.36	H-terminated 3H⁺
898.62629	3	$C_{135}H_{245}N_{27}O_{28}S_{0}H^{+}_{3}$	-0.26	H-terminated 3H⁺
931.64967	3	$C_{140}H_{254}N_{28}O_{29}S_{0}H^{+}_{3}$	0.37	H-terminated 3H⁺
964.67314	3	$C_{145}H_{263}N_{29}O_{30}S_{0}H^{+}_{3}$	1.04	H-terminated 3H⁺
997.69408	3	$C_{150}H_{272}N_{30}O_{31}S_{0}H^{+}_{3}$	-0.86	H-terminated 3H⁺
1030.71833	3	$C_{155}H_{281}N_{31}O_{32}S_{0}H^{+}_{3}$	0.57	H-terminated 3H⁺
1063.74081	3	$C_{160}H_{290}N_{32}O_{33}S_{0}H^{+}_{3}$	0.25	H-terminated 3H⁺
1096.76361	3	$C_{165}H_{299}N_{33}O_{34}S_{0}H^{+}_{3}$	0.24	H-terminated 3H⁺
1129.78694	3	$C_{170}H_{308}N_{34}O_{35}S_{0}H^{+}_{3}$	0.69	H-terminated 3H⁺
1162.80659	3	$C_{175}H_{317}N_{35}O_{36}S_{0}H^{+}_{3}$	-2.04	H-terminated 3H⁺
1195.83309	3	$C_{180}H_{326}N_{36}O_{37}S_{0}H^{+}_{3}$	1.11	H-terminated 3H⁺
797.15761	5	$C_{200}H_{362}N_{40}O_{41}S_{0}H^{+}_{5}$	1.14	H-terminated 5H⁺
816.97043	5	$C_{205}H_{371}N_{41}O_{42}S_{0}H^{+}_{5}$	0.06	H-terminated 5H⁺
836.784	5	$C_{210}H_{380}N_{42}O_{43}S_{0}H^{+}_{5}$	-0.08	H-terminated 5H⁺
856.59747	5	$C_{215}H_{389}N_{43}O_{44}S_{0}H^{+}_{5}$	-0.33	H-terminated 5H⁺
876.41085	5	$C_{220}H_{398}N_{44}O_{45}S_{0}H^{+}_{5}$	-0.66	H-terminated 5H⁺
896.22618	5	$C_{225}H_{407}N_{45}O_{46}S_{0}H^{+}_{5}$	1.19	H-terminated 5H⁺
916.03937	5	$C_{230}H_{416}N_{46}O_{47}S_{0}H^{+}_{5}$	0.63	H-terminated 5H⁺
935.85247	5	$C_{235}H_{425}N_{47}O_{48}S_{0}H^{+}_{5}$	-0.01	H-terminated 5H⁺
955.6663	5	$C_{240}H_{434}N_{48}O_{49}S_{0}H^{+}_{5}$	0.14	H-terminated 5H⁺
975.47982	5	$C_{245}H_{443}N_{49}O_{50}S_{0}H^{+}_{5}$	-0.03	H-terminated 5H⁺
995.29467	5	$C_{250}H_{452}N_{50}O_{51}S_{0}H^{+}_{5}$	1.15	H-terminated 5H⁺
1015.10531	5	$C_{255}H_{461}N_{51}O_{52}S_{0}H^{+}_{5}$	-1.87	H-terminated 5H⁺
1034.92091	5	$C_{260}H_{470}N_{52}O_{53}S_{0}H^{+}_{5}$	0.02	H-terminated 5H⁺
1054.73506	5	$C_{265}H_{479}N_{53}O_{54}S_{0}H^{+}_{5}$	0.46	H-terminated 5H⁺
1074.54768	5	$C_{270}H_{488}N_{54}O_{55}S_{0}H^{+}_{5}$	-0.54	H-terminated 5H⁺
1094.35955	5	$C_{275}H_{497}N_{55}O_{56}S_{0}H^{+}_{5}$	-2.19	H-terminated 5H⁺
1114.17697	5	$C_{280}H_{506}N_{56}O_{57}S_{0}H^{+}_{5}$	1.21	H-terminated 5H⁺
1133.98707	5	$C_{285}H_{515}N_{57}O_{58}S_{0}H^{+}_{5}$	-1.97	H-terminated 5H⁺
1153.80047	5	$C_{290}H_{524}N_{58}O_{59}S_{0}H^{+}_{5}$	-2.18	H-terminated 5H⁺
1173.61526	5	$C_{295}H_{533}N_{59}O_{60}S_{0}H^{+}_{5}$	-1.2	H-terminated 5H⁺
1193.42785	5	$C_{300}H_{542}N_{60}O_{61}S_{0}H^{+}_{5}$	-2.1	H-terminated 5H⁺
1233.05779	5	$C_{310}H_{560}N_{62}O_{63}S_{0}H^{+}_{5}$	0.06	H-terminated 5H⁺
1252.87239	5	$C_{315}H_{569}N_{63}O_{64}S_{0}H^{+}_{5}$	0.79	H-terminated 5H⁺
		Average	-1.42	

Table S 7: Cyclic peptide polymer conjugate IRMPD Figure 3B

m/z	charge	chemical formula	Error (ppm)	assignment
114.09137	1	$C_6H_{11}N_1O_1H+_1$	0.26	L (b/y)
129.10225	1	$C_6H_{12}N_2O_1H+_1$	0.08	K (b/y)
159.09169	1	$C_{10}H_{10}N_2O_0H+_1$	0.10	W (b/y) - CO
187.08662	1	$C_{11}H_{10}N_2O_1H+_1$	0.16	W (b/y)
242.18635	1	$C_{12}H_{23}N_3O_2H+_1$	0.19	LK (b/y)
300.17071	1	$C_{17}H_{21}N_3O_2H+_1$	0.19	WL (b/y)
355.27079	1	$C_{18}H_{34}N_4O_3H+_1$	1.19	LKL (b/y)
413.25476	1	$C_{23}H_{32}N_4O_3H+_1$	0.10	LWL (b/y)
428.26569	1	$C_{23}H_{33}N_5O_3H+_1$	0.17	KLW (b/y)
486.25008	1	$C_{28}H_{31}N_5O_3H+_1$	0.23	WLW (b/y)
541.34982	1	$C_{29}H_{44}N_6O_4H+_1$	0.26	LKLW (b/y)
599.33429	1	$C_{34}H_{42}N_6O_4H+_1$	0.43	WLWL (b/y)
654.4338	1	$C_{35}H_{55}N_7O_5H+_1$	0.09	LKLWL (b/y)
667.39666	1	$C_{39}H_{50}N_6O_4H+_1$	0.04	LWLWL (a/z) -H2
712.41823	1	$C_{40}H_{53}N_7O_5H+_1$	0.19	LWLWL (b/y)
727.42916	1	$C_{40}H_{54}N_8O_5H+_1$	0.23	KLWLW (b/y)
740.39257	1	$C_{44}H_{49}N_7O_4H+_1$	0.93	WLWLW (a/z) -H2
785.41313	1	$C_{45}H_{52}N_8O_5H+_1$	-0.27	WLWLW (b/y)
822.50254	1	$C_{46}H_{63}N_9O_5H+_1$	0.06	LKLWLW-H2O
840.51333	1	$C_{46}H_{65}N_9O_6H+_1$	0.32	WLKLWL (b/y)
853.47564	1	$C_{50}H_{60}N_8O_5H+_1$	-0.36	LWLWLW (a/z) -H2
880.48694	1	$C_{51}H_{61}N_9O_5H+_1$	0.11	LWLWLW-H2O
898.49723	1	$C_{51}H_{63}N_9O_6H+_1$	-0.20	LWLWLW (b/y)
953.5976	1	$C_{52}H_{76}N_{10}O_7H+_1$	0.50	LKLWLWL (b/y)
1011.58238	1	$C_{57}H_{74}N_{10}O_7H+_1$	0.90	LWLWLWL (b/y)
1026.59305	1	$C_{57}H_{75}N_{11}O_7H+_1$	0.66	WLKLWLW (b/y)
1139.6768	1	$C_{63}H_{86}N_{12}O_8H+_1$	0.32	cypep
100.07572	1	$C_5H_9N_1O_1H^+_1$	0.30	1-Internal
199.14409	1	$C_{10}H_{18}N_2O_2H^+_1$	-0.07	1-Internal
298.21256	1	$C_{15}H_{27}N_3O_3H^+_1$	0.14	1-Internal
397.281	1	$C_{20}H_{36}N_4O_4H^+_1$	0.17	1-Internal
496.34944	1	$C_{25}H_{45}N_5O_5H^+_1$	0.19	1-Internal
595.41784	1	$C_{30}H_{54}N_6O_6H^+_1$	0.13	1-Internal
694.4863	1	$C_{35}H_{63}N_7O_7H^+_1$	0.18	1-Internal
793.55471	1	$C_{40}H_{72}N_8O_8H^+_1$	0.15	1-Internal
892.62325	1	$C_{45}H_{81}N_9O_9H^+_1$	0.28	1-Internal
991.69156	1	$C_{50}H_{90}N_{10}O_{10}H^+_1$	0.15	1-Internal
1090.7601	1	$C_{55}H_{99}N_{11}O_{11}H^+_1$	0.25	1-Internal
1189.82837	1	$C_{60}H_{108}N_{12}O_{12}H^+_1$	0.11	1-Internal
1288.89621	1	$C_{65}H_{117}N_{13}O_{13}H^+_1$	-0.35	1-Internal
1387.96509	1	$C_{70}H_{126}N_{14}O_{14}H^+_1$	0.01	1-Internal
1487.03249	1	$C_{75}H_{135}N_{15}O_{15}H^+_1$	-0.67	1-Internal
1586.1007	1	$C_{80}H_{144}N_{16}O_{16}H^+_1$	-0.76	1-Internal
1685.16979	1	$C_{85}H_{153}N_{17}O_{17}H^+_1$	-0.31	1-Internal
1784.23723	1	$C_{90}H_{162}N_{18}O_{18}H^+_1$	-0.84	1-Internal
1883.3066	1	$C_{95}H_{171}N_{19}O_{19}H^+_1$	-0.29	1-Internal
1982.37387	1	$C_{100}H_{180}N_{20}O_{20}H^+_1$	-0.85	1-Internal

2081.44084	1	$C_{105}H_{189}N_{21}O_{21}H^{+}_{1}$	-1.50	1-Internal
2180.51309	1	$C_{110}H_{198}N_{22}O_{22}H^{+}_{1}$	0.32	1-Internal
2279.58221	1	$C_{115}H_{207}N_{23}O_{23}H^{+}_{1}$	0.62	1-Internal
2378.64738	1	$C_{120}H_{216}N_{24}O_{24}H^{+}_{1}$	-0.77	1-Internal
2477.71743	1	$C_{125}H_{225}N_{25}O_{25}H^{+}_{1}$	-0.08	1-Internal
162.61777	2	$C_{17}H_{29}N_{3}O_{3}H^{+}_{2}$	0.29	2-Internal
212.15195	2	$C_{22}H_{38}N_{4}O_{4}H^{+}_{2}$	0.10	2-Internal
261.68618	2	$C_{27}H_{47}N_{5}O_{5}H^{+}_{2}$	0.17	2-Internal
311.22042	2	$C_{32}H_{56}N_{6}O_{6}H^{+}_{2}$	0.25	2-Internal
360.75463	2	$C_{37}H_{65}N_{7}O_{7}H^{+}_{2}$	0.22	2-Internal
410.28883	2	$C_{42}H_{74}N_{8}O_{8}H^{+}_{2}$	0.18	2-Internal
459.82302	2	$C_{47}H_{83}N_{9}O_{9}H^{+}_{2}$	0.12	2-Internal
509.35725	2	$C_{52}H_{92}N_{10}O_{10}H^{+}_{2}$	0.15	2-Internal
558.89145	2	$C_{57}H_{101}N_{11}O_{11}H^{+}_{2}$	0.13	2-Internal
608.42566	2	$C_{62}H_{110}N_{12}O_{12}H^{+}_{2}$	0.12	2-Internal
657.95993	2	$C_{67}H_{119}N_{13}O_{13}H^{+}_{2}$	0.21	2-Internal
707.4941	2	$C_{72}H_{128}N_{14}O_{14}H^{+}_{2}$	0.14	2-Internal
757.02835	2	$C_{77}H_{137}N_{15}O_{15}H^{+}_{2}$	0.19	2-Internal
806.56235	2	$C_{82}H_{146}N_{16}O_{16}H^{+}_{2}$	-0.08	2-Internal
856.09668	2	$C_{87}H_{155}N_{17}O_{17}H^{+}_{2}$	0.07	2-Internal
		Abs Average	0.31	

Fragment	Initial fragment mass	Error (ppm)	Ref Code (table above)
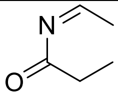	100.07572	0.3	1
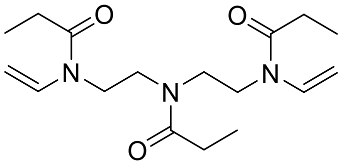	162.61777 (2+)	0.3	2

Table S 8: Cyclic peptide polymer conjugate ECD Figure 3D

mz	Charge	Chemical Formula	Error (ppm)	Assignment
217.15467	1	$C_{10}H_{20}N_2O_3H^+_1$	0.00	a_2
316.22305	1	$C_{15}H_{29}N_3O_4H^+_1$	-0.10	a_3
415.29142	1	$C_{20}H_{38}N_4O_5H^+_1$	-0.18	a_4
514.35985	1	$C_{25}H_{47}N_5O_6H^+_1$	-0.12	a_5
613.42834	1	$C_{30}H_{56}N_6O_7H^+_1$	0.03	a_6
712.49665	1	$C_{35}H_{65}N_7O_8H^+_1$	-0.12	a_7
811.56518	1	$C_{40}H_{74}N_8O_9H^+_1$	0.03	a_8
910.63372	1	$C_{45}H_{83}N_9O_{10}H^+_1$	0.17	a_9
1009.70221	1	$C_{50}H_{92}N_{10}O_{11}H^+_1$	0.23	a_{10}
1108.77042	1	$C_{55}H_{101}N_{11}O_{12}H^+_1$	0.02	a_{11}
1207.83952	1	$C_{60}H_{110}N_{12}O_{13}H^+_1$	0.59	a_{12}
1306.90778	1	$C_{65}H_{119}N_{13}O_{14}H^+_1$	0.43	a_{13}
1405.97637	1	$C_{70}H_{128}N_{14}O_{15}H^+_1$	0.52	a_{14}
1505.04344	1	$C_{75}H_{137}N_{15}O_{16}H^+_1$	-0.41	a_{15}
1604.11303	1	$C_{80}H_{146}N_{16}O_{17}H^+_1$	0.35	a_{16}
1703.18068	1	$C_{85}H_{155}N_{17}O_{18}H^+_1$	-0.12	a_{17}
1802.24818	1	$C_{90}H_{164}N_{18}O_{19}H^+_1$	-0.62	a_{18}
1901.31778	1	$C_{95}H_{173}N_{19}O_{20}H^+_1$	0.04	a_{19}
2000.38413	1	$C_{100}H_{182}N_{20}O_{21}H^+_1$	-0.99	a_{20}
2099.45424	1	$C_{105}H_{191}N_{21}O_{22}H^+_1$	-0.14	a_{21}
2198.51471	1	$C_{110}H_{200}N_{22}O_{23}H^+_1$	-3.75	a_{22}
2297.59648	1	$C_{115}H_{209}N_{23}O_{24}H^+_1$	2.23	a_{23}
2396.65113	1	$C_{120}H_{218}N_{24}O_{25}H^+_1$	-3.61	a_{24}
1072.67326	2	$C_{113}H_{174}N_{22}O_{19}H^+_2$	-0.28	z_9
1122.20684	2	$C_{118}H_{183}N_{23}O_{20}H^+_2$	-0.82	z_{10}
1171.74088	2	$C_{123}H_{192}N_{24}O_{21}H^+_2$	-0.93	z_{11}
1221.27591	2	$C_{128}H_{201}N_{25}O_{22}H^+_2$	-0.22	z_{12}
1270.8089	2	$C_{133}H_{210}N_{26}O_{23}H^+_2$	-1.17	z_{13}
1320.34401	2	$C_{138}H_{219}N_{27}O_{24}H^+_2$	-0.44	z_{14}
1369.87803	2	$C_{143}H_{228}N_{28}O_{25}H^+_2$	-0.56	z_{15}
1419.41092	2	$C_{148}H_{237}N_{29}O_{26}H^+_2$	-1.47	z_{16}
1468.94627	2	$C_{153}H_{246}N_{30}O_{27}H^+_2$	-0.64	z_{17}
1518.47992	2	$C_{158}H_{255}N_{31}O_{28}H^+_2$	-0.99	z_{18}
1617.54842	2	$C_{168}H_{273}N_{33}O_{30}H^+_2$	-0.87	z_{20}
1667.08212	2	$C_{173}H_{282}N_{34}O_{31}H^+_2$	-1.15	z_{21}
1716.61699	2	$C_{178}H_{291}N_{35}O_{32}H^+_2$	-0.73	z_{22}
1766.15243	2	$C_{183}H_{300}N_{36}O_{33}H^+_2$	-0.01	z_{23}
1815.68703	2	$C_{188}H_{309}N_{37}O_{34}H^+_2$	0.20	z_{24}
1865.2174	2	$C_{193}H_{318}N_{38}O_{35}H^+_2$	-1.86	z_{25}
1914.74772	2	$C_{198}H_{327}N_{39}O_{36}H^+_2$	-3.84	z_{26}
1964.28914	2	$C_{203}H_{336}N_{40}O_{37}H^+_2$	-0.07	z_{27}
2013.81841	2	$C_{208}H_{345}N_{41}O_{38}H^+_2$	-2.52	z_{28}
1351.7913	1	$C_{73}H_{102}N_{14}O_{11}H^+_1$	-0.91	z_2
1450.86469	1	$C_{78}H_{111}N_{15}O_{12}H^+_1$	2.58	z_3
1648.99588	1	$C_{88}H_{129}N_{17}O_{14}H^+_1$	-1.15	z_5
1946.20106	1	$C_{103}H_{156}N_{20}O_{17}H^+_1$	-1.00	z_7

2045.27384	1	$C_{108}H_{165}N_{21}O_{18}H^{+}_{1}$	1.18	z_{8}
2144.33867	1	$C_{113}H_{174}N_{22}O_{19}H^{+}_{1}$	-0.54	z_{9}
2342.47733	1	$C_{123}H_{192}N_{24}O_{21}H^{+}_{1}$	0.28	z_{11}
653.95716	2	$C_{65}H_{119}N_{13}O_{14}H^{+}_{2}$	-0.14	a_{13}
703.49161	2	$C_{70}H_{128}N_{14}O_{15}H^{+}_{2}$	0.22	a_{14}
753.02567	2	$C_{75}H_{137}N_{15}O_{16}H^{+}_{2}$	0.01	a_{15}
802.55992	2	$C_{80}H_{146}N_{16}O_{17}H^{+}_{2}$	0.06	a_{16}
852.09448	2	$C_{85}H_{155}N_{17}O_{18}H^{+}_{2}$	0.47	a_{17}
901.62841	2	$C_{90}H_{164}N_{18}O_{19}H^{+}_{2}$	0.14	a_{18}
951.16277	2	$C_{95}H_{173}N_{19}O_{20}H^{+}_{2}$	0.29	a_{19}
1000.69679	2	$C_{100}H_{182}N_{20}O_{21}H^{+}_{2}$	0.09	a_{20}
1050.23099	2	$C_{105}H_{191}N_{21}O_{22}H^{+}_{2}$	0.08	a_{21}
1099.76537	2	$C_{110}H_{200}N_{22}O_{23}H^{+}_{2}$	0.23	a_{22}
1149.29948	2	$C_{115}H_{209}N_{23}O_{24}H^{+}_{2}$	0.14	a_{23}
1198.83355	2	$C_{120}H_{218}N_{24}O_{25}H^{+}_{2}$	0.02	a_{24}
1297.901	2	$C_{130}H_{236}N_{26}O_{27}H^{+}_{2}$	-0.72	a_{26}
1347.43518	2	$C_{135}H_{245}N_{27}O_{28}H^{+}_{2}$	-0.72	a_{27}
1396.96947	2	$C_{140}H_{254}N_{28}O_{29}H^{+}_{2}$	-0.63	a_{28}
1446.50402	2	$C_{145}H_{263}N_{29}O_{30}H^{+}_{2}$	-0.37	a_{29}
1496.03889	2	$C_{150}H_{272}N_{30}O_{31}H^{+}_{2}$	0.08	a_{30}
1545.57296	2	$C_{155}H_{281}N_{31}O_{32}H^{+}_{2}$	-0.01	a_{31}
1595.10537	2	$C_{160}H_{290}N_{32}O_{33}H^{+}_{2}$	-1.14	a_{32}
1644.64191	2	$C_{165}H_{299}N_{33}O_{34}H^{+}_{2}$	0.32	a_{33}
1694.17383	2	$C_{170}H_{308}N_{34}O_{35}H^{+}_{2}$	-1.04	a_{34}
1743.70726	2	$C_{175}H_{317}N_{35}O_{36}H^{+}_{2}$	-1.46	a_{35}
1793.2411	2	$C_{180}H_{326}N_{36}O_{37}H^{+}_{2}$	-1.62	a_{36}
1842.77537	2	$C_{185}H_{335}N_{37}O_{38}H^{+}_{2}$	-1.54	a_{37}
1892.31328	2	$C_{190}H_{344}N_{38}O_{39}H^{+}_{2}$	0.45	a_{38}
1941.8461	2	$C_{195}H_{353}N_{39}O_{40}H^{+}_{2}$	-0.27	a_{39}
2040.91835	2	$C_{205}H_{371}N_{41}O_{42}H^{+}_{2}$	1.62	a_{41}
2090.44512	2	$C_{210}H_{380}N_{42}O_{43}H^{+}_{2}$	-1.98	a_{42}
2139.98718	2	$C_{215}H_{389}N_{43}O_{44}H^{+}_{2}$	1.74	a_{43}
2189.51765	2	$C_{220}H_{398}N_{44}O_{45}H^{+}_{2}$	-0.01	a_{44}
2288.58794	2	$C_{230}H_{416}N_{46}O_{47}H^{+}_{2}$	0.81	a_{46}
2338.12616	2	$C_{235}H_{425}N_{47}O_{48}H^{+}_{2}$	2.51	a_{47}
201.15976	1	$C_{10}H_{20}N_{2}O_{2}H^{+}_{1}$	0.03	1-Internal
300.22805	1	$C_{15}H_{29}N_{3}O_{3}H^{+}_{1}$	-0.39	1-Internal
399.29651	1	$C_{20}H_{38}N_{4}O_{4}H^{+}_{1}$	-0.18	1-Internal
498.36491	1	$C_{25}H_{47}N_{5}O_{5}H^{+}_{1}$	-0.17	1-Internal
597.43339	1	$C_{30}H_{56}N_{6}O_{6}H^{+}_{1}$	-0.03	1-Internal
696.50167	1	$C_{35}H_{65}N_{7}O_{7}H^{+}_{1}$	-0.22	1-Internal
795.57018	1	$C_{40}H_{74}N_{8}O_{8}H^{+}_{1}$	-0.07	1-Internal
894.63869	1	$C_{45}H_{83}N_{9}O_{9}H^{+}_{1}$	0.04	1-Internal
993.7071	1	$C_{50}H_{92}N_{10}O_{10}H^{+}_{1}$	0.03	1-Internal
1092.77594	1	$C_{55}H_{101}N_{11}O_{11}H^{+}_{1}$	0.42	1-Internal
1389.98154	1	$C_{70}H_{128}N_{14}O_{14}H^{+}_{1}$	0.59	1-Internal
1489.04637	1	$C_{75}H_{137}N_{15}O_{15}H^{+}_{1}$	-1.86	1-Internal
1588.11559	1	$C_{80}H_{146}N_{16}O_{16}H^{+}_{1}$	-1.23	1-Internal
272.19683	1	$C_{13}H_{25}N_{3}O_{3}H^{+}_{1}$	-0.14	2-Internal

371.26531	1	C18H34N4O4H^{+}1	0.08	2-Internal
470.33361	1	C23H43N5O5H^{+}1	-0.18	2-Internal
569.402	1	C28H52N6O6H^{+}1	-0.19	2-Internal
668.47055	1	C33H61N7O7H^{+}1	0.04	2-Internal
767.5389	1	C38H70N8O8H^{+}1	-0.05	2-Internal
866.60699	1	C43H79N9O9H^{+}1	-0.42	2-Internal
965.67582	1	C48H88N10O10H^{+}1	0.06	2-Internal
1064.74425	1	C53H97N11O11H^{+}1	0.07	2-Internal
1163.81228	1	C58H106N12O12H^{+}1	-0.27	2-Internal
1262.88108	1	C63H115N13O13H^{+}1	0.06	2-Internal
1361.94886	1	C68H124N14O14H^{+}1	-0.41	2-Internal
1461.01589	1	C73H133N15O15H^{+}1	-1.33<br>1	2-Internal

Fragment	Initial fragment mass	Error (ppm)	Ref Code (table above)
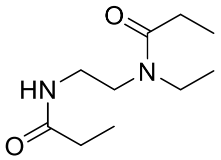	201.15976	0.0	1
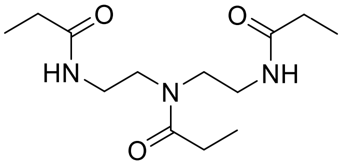	272.19683	-0.1	2

Table S 9: Cyclic peptide polymer conjugate UVPD Figure 4A

m/z	charge	Chemical formula	Error (ppm)	assignment
114.0914	1	$C_6H_{11}N_1O_1H^+_1$	0.52	L (b/y)
129.1023	1	$C_6H_{12}N_2O_1H^+_1$	0.47	K (b/y)
242.18633	1	$C_{12}H_{23}N_3O_2H^+_1$	0.11	LK (b/y)
300.1707	1	$C_{17}H_{21}N_3O_2H^+_1$	0.16	WL (b/y)
355.27031	1	$C_{18}H_{34}N_4O_3H^+_1$	-0.16	LKL (b/y)
413.25472	1	$C_{23}H_{32}N_4O_3H^+_1$	0.01	LWL (b/y)
428.26574	1	$C_{23}H_{33}N_5O_3H^+_1$	0.29	KLW (b/y)
486.25007	1	$C_{28}H_{31}N_5O_3H^+_1$	0.21	WLW (b/y)
541.34968	1	$C_{29}H_{44}N_6O_4H^+_1$	0.00	LKLW (b/y)
599.33417	1	$C_{34}H_{42}N_6O_4H^+_1$	0.23	WLWL (b/y)
654.43386	1	$C_{35}H_{55}N_7O_5H^+_1$	0.18	LKLWL (b/y)
712.41849	1	$C_{40}H_{53}N_7O_5H^+_1$	0.56	LWLWL (b/y)
727.42984	1	$C_{40}H_{54}N_8O_5H^+_1$	1.16	WLKLW (b/y)
785.41366	1	$C_{45}H_{52}N_8O_5H^+_1$	0.40	WLWLW (b/y)
840.51319	1	$C_{46}H_{65}N_9O_6H^+_1$	0.16	WLKLWL (b/y)
898.49799	1	$C_{51}H_{63}N_9O_6H^+_1$	0.65	WLWLWL (b/y)
953.59757	1	$C_{52}H_{76}N_{10}O_7H^+_1$	0.47	LWLKLWL (b/y)
1011.58252	1	$C_{57}H_{74}N_{10}O_7H^+_1$	1.04	LWLWLWL (b/y)
1026.59239	1	$C_{57}H_{75}N_{11}O_7H^+_1$	0.02	WLKLWLW (b/y)
1139.67718	1	$C_{63}H_{86}N_{12}O_8H^+_1$	0.65	Cycpep

337.25974	1	$C_{18}H_{32}N_4O_2H^+_1$	-0.19	LKL-H_2O
523.33895	1	$C_{29}H_{42}N_6O_3H^+_1$	-0.32	LKLW-H_2O
255.14918	1	$C_{16}H_{18}N_2O_1H^+_1$	-0.04	WL (a/z) -H_2
554.31271	1	$C_{33}H_{39}N_5O_3H^+_1$	0.26	WL (c/y) +H
217.15469	1	$C_{10}H_{20}N_2O_3H^+_1$	0.10	a_2
316.22314	1	$C_{15}H_{29}N_3O_4H^+_1$	0.18	a_3
415.29153	1	$C_{20}H_{38}N_4O_5H^+_1$	0.08	a_8
514.36005	1	$C_{25}H_{47}N_5O_6H^+_1$	0.27	a_5
613.42848	1	$C_{30}H_{56}N_6O_7H^+_1$	0.25	a_6
712.49694	1	$C_{35}H_{65}N_7O_8H^+_1$	0.28	a_7
811.56528	1	$C_{40}H_{74}N_8O_9H^+_1$	0.16	a_8
910.63377	1	$C_{45}H_{83}N_9O_{10}H^+_1$	0.22	a_9
1009.70225	1	$C_{50}H_{92}N_{10}O_{11}H^+_1$	0.27	a_{10}
1108.77104	1	$C_{55}H_{101}N_{11}O_{12}H^+_1$	0.58	a_{11}
1207.83993	1	$C_{60}H_{110}N_{12}O_{13}H^+_1$	0.93	a_{12}
1306.90838	1	$C_{65}H_{119}N_{13}O_{14}H^+_1$	0.89	a_{13}
1405.97682	1	$C_{70}H_{128}N_{14}O_{15}H^+_1$	0.84	a_{14}
1505.04381	1	$C_{75}H_{137}N_{15}O_{16}H^+_1$	-0.16	a_{15}
1604.11281	1	$C_{80}H_{146}N_{16}O_{17}H^+_1$	0.22	a_{16}
1703.17823	1	$C_{85}H_{155}N_{17}O_{18}H^+_1$	-1.55	a_{17}
406.28623	2	$C_{40}H_{74}N_8O_9H^+_2$	0.04	a_4
455.82042	2	$C_{45}H_{83}N_9O_{10}H^+_2$	0.00	a_5
505.35468	2	$C_{50}H_{92}N_{10}O_{11}H^+_2$	0.10	a_6
554.8889	2	$C_{55}H_{101}N_{11}O_{12}H^+_2$	0.12	a_7
604.42316	2	$C_{60}H_{110}N_{12}O_{13}H^+_2$	0.19	a_8
653.95746	2	$C_{65}H_{119}N_{13}O_{14}H^+_2$	0.32	a_9
703.49178	2	$C_{70}H_{128}N_{14}O_{15}H^+_2$	0.46	a_{14}
753.02594	2	$C_{75}H_{137}N_{15}O_{16}H^+_2$	0.37	a_{15}
802.5602	2	$C_{80}H_{146}N_{16}O_{17}H^+_2$	0.41	a_{16}
852.09452	2	$C_{85}H_{155}N_{17}O_{18}H^+_2$	0.52	a_{17}
901.6286	2	$C_{90}H_{164}N_{18}O_{19}H^+_2$	0.35	a_{18}
951.16302	2	$C_{95}H_{173}N_{19}O_{20}H^+_2$	0.56	a_{19}
1000.69726	2	$C_{100}H_{182}N_{20}O_{21}H^+_2$	0.56	a_{20}
1050.23124	2	$C_{105}H_{191}N_{21}O_{22}H^+_2$	0.32	a_{21}
1099.76592	2	$C_{110}H_{200}N_{22}O_{23}H^+_2$	0.73	a_{22}
1149.2999	2	$C_{115}H_{209}N_{23}O_{24}H^+_2$	0.51	a_{23}
1198.83358	2	$C_{120}H_{218}N_{24}O_{25}H^+_2$	0.05	a_{24}
1297.90129	2	$C_{130}H_{236}N_{26}O_{27}H^+_2$	-0.50	a_{26}
1347.43543	2	$C_{135}H_{245}N_{27}O_{28}H^+_2$	-0.53	a_{27}
1396.96982	2	$C_{140}H_{254}N_{28}O_{29}H^+_2$	-0.38	a_{28}
1446.50538	2	$C_{145}H_{263}N_{29}O_{30}H^+_2$	0.57	a_{29}
1496.04154	2	$C_{150}H_{272}N_{30}O_{31}H^+_2$	1.85	a_{30}
1545.57474	2	$C_{155}H_{281}N_{31}O_{32}H^+_2$	1.14	a_{31}
1595.10638	2	$C_{160}H_{290}N_{32}O_{33}H^+_2$	-0.50	a_{32}
667.46724	3	$C_{100}H_{182}N_{20}O_{21}H^+_3$	0.52	a_{20}
700.48976	3	$C_{105}H_{191}N_{21}O_{22}H^+_3$	0.09	a_{21}
766.53573	3	$C_{115}H_{209}N_{23}O_{24}H^+_3$	0.55	a_{23}
799.55837	3	$C_{120}H_{218}N_{24}O_{25}H^+_3$	0.33	a_{24}
832.58119	3	$C_{125}H_{227}N_{25}O_{26}H^+_3$	0.33	a_{25}

898.62633	3	$C_{135}H_{245}N_{27}O_{28}H^{+}_{3}$	-0.22	a_{27}
931.64939	3	$C_{140}H_{254}N_{28}O_{29}H^{+}_{3}$	0.07	a_{28}
997.69468	3	$C_{150}H_{272}N_{30}O_{31}H^{+}_{3}$	-0.26	a_{30}
1030.71759	3	$C_{155}H_{281}N_{31}O_{32}H^{+}_{3}$	-0.15	a_{31}
1096.76311	3	$C_{165}H_{299}N_{33}O_{34}H^{+}_{3}$	-0.22	a_{33}
1129.78621	3	$C_{170}H_{308}N_{34}O_{35}H^{+}_{3}$	0.05	a_{34}
1195.83175	3	$C_{180}H_{326}N_{36}O_{37}H^{+}_{3}$	-0.01	a_{36}
1228.85518	3	$C_{185}H_{335}N_{37}O_{38}H^{+}_{3}$	0.50	a_{37}
1294.90104	3	$C_{195}H_{353}N_{39}O_{40}H^{+}_{3}$	0.66	a_{39}
1327.92278	3	$C_{200}H_{362}N_{40}O_{41}H^{+}_{3}$	-0.15	a_{40}
1393.96908	3	$C_{210}H_{380}N_{42}O_{43}H^{+}_{3}$	0.35	a_{42}
1426.99163	3	$C_{215}H_{389}N_{43}O_{44}H^{+}_{3}$	0.16	a_{43}
1493.03901	3	$C_{225}H_{407}N_{45}O_{46}H^{+}_{3}$	1.34	a_{45}
996.19392	4	$C_{200}H_{362}N_{40}O_{41}H^{+}_{4}$	-0.14	a_{40}
1070.49587	4	$C_{215}H_{389}N_{43}O_{44}H^{+}_{4}$	0.47	a_{43}
1095.26172	4	$C_{220}H_{398}N_{44}O_{45}H^{+}_{4}$	-0.69	a_{44}
1144.79683	4	$C_{230}H_{416}N_{46}O_{47}H^{+}_{4}$	0.13	a_{46}
1169.56357	4	$C_{235}H_{425}N_{47}O_{48}H^{+}_{4}$	-0.18	a_{47}
1194.33003	4	$C_{240}H_{434}N_{48}O_{49}H^{+}_{4}$	-0.72	a_{48}
1268.63269	4	$C_{255}H_{461}N_{51}O_{52}H^{+}_{4}$	0.39	a_{51}
1568.01618	2	$C_{163}H_{264}N_{32}O_{29}H^{+}_{2}$	0.35	x_{20}
1617.54784	2	$C_{168}H_{273}N_{33}O_{30}H^{+}_{2}$	-1.23	x_{21}
1667.08334	2	$C_{173}H_{282}N_{34}O_{31}H^{+}_{2}$	-0.42	x_{22}
1716.61913	2	$C_{178}H_{291}N_{35}O_{32}H^{+}_{2}$	0.51	x_{23}
1766.15317	2	$C_{183}H_{300}N_{36}O_{33}H^{+}_{2}$	0.41	x_{24}
1815.68781	2	$C_{188}H_{309}N_{37}O_{34}H^{+}_{2}$	0.63	x_{25}
1865.21794	2	$C_{193}H_{318}N_{38}O_{35}H^{+}_{2}$	-1.57	x_{26}
1914.75104	2	$C_{198}H_{327}N_{39}O_{36}H^{+}_{2}$	-2.11	x_{27}
1964.29052	2	$C_{203}H_{336}N_{40}O_{37}H^{+}_{2}$	0.63	x_{28}
2013.82005	2	$C_{208}H_{345}N_{41}O_{38}H^{+}_{2}$	-1.71	x_{29}
2063.35857	2	$C_{213}H_{354}N_{42}O_{39}H^{+}_{2}$	0.42	x_{30}
1276.838	3	$C_{198}H_{327}N_{39}O_{36}H^{+}_{3}$	-0.89	x_{27}
1309.86268	3	$C_{203}H_{336}N_{40}O_{37}H^{+}_{3}$	0.56	x_{28}
1342.88351	3	$C_{208}H_{345}N_{41}O_{38}H^{+}_{3}$	-0.92	x_{29}
1375.90715	3	$C_{213}H_{354}N_{42}O_{39}H^{+}_{3}$	-0.30	x_{30}
1408.93121	3	$C_{218}H_{363}N_{43}O_{40}H^{+}_{3}$	0.60	z_{31}
1474.97611	3	$C_{228}H_{381}N_{45}O_{42}H^{+}_{3}$	0.09	x_{33}
1507.99728	3	$C_{233}H_{390}N_{46}O_{43}H^{+}_{3}$	-0.99	x_{34}
1541.02115	3	$C_{238}H_{399}N_{47}O_{44}H^{+}_{3}$	-0.28	x_{35}
1574.04528	3	$C_{243}H_{408}N_{48}O_{45}H^{+}_{3}$	0.57	x_{36}
1607.06807	3	$C_{248}H_{417}N_{49}O_{46}H^{+}_{3}$	0.55	x_{37}
1640.09151	3	$C_{253}H_{426}N_{50}O_{47}H^{+}_{3}$	0.92	x_{38}
1673.11219	3	$C_{258}H_{435}N_{51}O_{48}H^{+}_{3}$	-0.36	x_{39}
1706.13262	3	$C_{263}H_{444}N_{52}O_{49}H^{+}_{3}$	-1.75	x_{40}
1739.15654	3	$C_{268}H_{453}N_{53}O_{50}H^{+}_{3}$	-1.07	x_{41}
1772.18462	3	$C_{273}H_{462}N_{54}O_{51}H^{+}_{3}$	1.92	x_{42}
1180.78699	4	$C_{243}H_{408}N_{48}O_{45}H^{+}_{4}$	1.59	x_{36}
1205.55168	4	$C_{248}H_{417}N_{49}O_{46}H^{+}_{4}$	-0.44	x_{37}
1279.85335	4	$C_{263}H_{444}N_{52}O_{49}H^{+}_{4}$	-0.13	x_{40}

1304.6199	4	$C_{268}H_{453}N_{53}O_{50}H^{+}_{4}$	-0.56	x_{41}
1329.38647	4	$C_{273}H_{462}N_{54}O_{51}H^{+}_{4}$	-0.95	x_{42}
1354.15504	4	$C_{278}H_{471}N_{55}O_{52}H^{+}_{4}$	0.15	x_{43}
1378.92063	4	$C_{283}H_{480}N_{56}O_{53}H^{+}_{4}$	-0.95	x_{44}
1403.68773	4	$C_{288}H_{489}N_{57}O_{54}H^{+}_{4}$	-0.93	x_{45}
1428.4565	4	$C_{293}H_{498}N_{58}O_{55}H^{+}_{4}$	0.25	x_{46}
1453.22314	4	$C_{298}H_{507}N_{59}O_{56}H^{+}_{4}$	-0.07	x_{47}
1477.98927	4	$C_{303}H_{516}N_{60}O_{57}H^{+}_{4}$	-0.73	x_{48}
1296.86575	4	$C_{267}H_{450}N_{53}O_{49}H^{+}_{4}$	-0.21	x_{41}
1321.63303	4	$C_{272}H_{459}N_{54}O_{50}H^{+}_{4}$	-0.08	x_{42}
1346.3988	4	$C_{277}H_{468}N_{55}O_{51}H^{+}_{4}$	-1.07	x_{43}
1371.16639	4	$C_{282}H_{477}N_{56}O_{52}H^{+}_{4}$	-0.69	x_{44}
1395.93267	4	$C_{287}H_{486}N_{57}O_{53}H^{+}_{4}$	-1.27	x_{45}
1420.69899	4	$C_{292}H_{495}N_{58}O_{54}H^{+}_{4}$	-1.80	x_{46}
1445.46608	4	$C_{297}H_{504}N_{59}O_{55}H^{+}_{4}$	-1.78	x_{47}
1470.2354	4	$C_{302}H_{513}N_{60}O_{56}H^{+}_{4}$	-0.24	x_{48}
1495.00257	4	$C_{307}H_{522}N_{61}O_{57}H^{+}_{4}$	-0.19	x_{49}
1169.77826	4	$C_{241}H_{404}N_{48}O_{44}H^{+}_{4}$	-0.25	x_{36}
1194.54551	4	$C_{246}H_{413}N_{49}O_{45}H^{+}_{4}$	-0.12	x_{37}
1219.31361	4	$C_{251}H_{422}N_{50}O_{46}H^{+}_{4}$	0.70	x_{38}
1268.84649	4	$C_{261}H_{440}N_{52}O_{48}H^{+}_{4}$	-0.38	x_{40}
1293.6141	4	$C_{266}H_{449}N_{53}O_{49}H^{+}_{4}$	0.02	x_{41}
1318.37891	4	$C_{271}H_{458}N_{54}O_{50}H^{+}_{4}$	-1.72	x_{42}
1343.14784	4	$C_{276}H_{467}N_{55}O_{51}H^{+}_{4}$	-0.33	x_{43}
1367.91389	4	$C_{281}H_{476}N_{56}O_{52}H^{+}_{4}$	-1.09	x_{44}
1392.68249	4	$C_{286}H_{485}N_{57}O_{53}H^{+}_{4}$	0.00	x_{45}
1417.4478	4	$C_{291}H_{494}N_{58}O_{54}H^{+}_{4}$	-1.26	x_{46}
1442.21072	4	$C_{296}H_{503}N_{59}O_{55}H^{+}_{4}$	-4.14	x_{47}
1466.98268	4	$C_{301}H_{512}N_{60}O_{56}H^{+}_{4}$	-0.76	x_{48}
341.25478	1	$C_{17}H_{32}N_{4}O_{3}H^{+}_{1}$	0.18	1-Internal
440.32322	1	$C_{22}H_{41}N_{5}O_{4}H^{+}_{1}$	0.20	1-Internal
539.39163	1	$C_{27}H_{50}N_{6}O_{5}H^{+}_{1}$	0.16	1-Internal
638.46009	1	$C_{32}H_{59}N_{7}O_{6}H^{+}_{1}$	0.21	1-Internal
836.59728	1	$C_{42}H_{77}N_{9}O_{8}H^{+}_{1}$	0.59	1-Internal
1034.73461	1	$C_{52}H_{95}N_{11}O_{10}H^{+}_{1}$	0.96	1-Internal
1133.80397	1	$C_{57}H_{104}N_{12}O_{11}H^{+}_{1}$	1.71	1-Internal
1232.87379	1	$C_{62}H_{113}N_{13}O_{12}H^{+}_{1}$	2.72	1-Internal
1629.14437	1	$C_{82}H_{149}N_{17}O_{16}H^{+}_{1}$	0.17	1-Internal
1728.21554	1	$C_{87}H_{158}N_{18}O_{17}H^{+}_{1}$	1.75	1-Internal
1827.28322	1	$C_{92}H_{167}N_{19}O_{18}H^{+}_{1}$	1.26	1-Internal
1926.35247	1	$C_{97}H_{176}N_{20}O_{19}H^{+}_{1}$	1.62	1-Internal
2025.41443	1	$C_{102}H_{185}N_{21}O_{20}H^{+}_{1}$	-1.64	1-Internal
199.14414	1	$C_{10}H_{18}N_{2}O_{2}H^{+}_{1}$	0.18	2-Internal
298.21252	1	$C_{15}H_{27}N_{3}O_{3}H^{+}_{1}$	0.01	2-Internal
397.281	1	$C_{20}H_{36}N_{4}O_{4}H^{+}_{1}$	0.17	2-Internal
496.34941	1	$C_{25}H_{45}N_{5}O_{5}H^{+}_{1}$	0.13	2-Internal
595.41788	1	$C_{30}H_{54}N_{6}O_{6}H^{+}_{1}$	0.20	2-Internal
892.62343	1	$C_{45}H_{81}N_{9}O_{9}H^{+}_{1}$	0.48	2-Internal
1090.7606	1	$C_{55}H_{99}N_{11}O_{11}H^{+}_{1}$	0.71	2-Internal

1387.96586	1	$C_{70}H_{126}N_{14}O_{14}H^{+}_{1}$	0.57	2-Internal
1487.03271	1	$C_{75}H_{135}N_{15}O_{15}H^{+}_{1}$	-0.52	2-Internal
1586.10216	1	$C_{80}H_{144}N_{16}O_{16}H^{+}_{1}$	0.16	2-Internal
1982.37456	1	$C_{100}H_{180}N_{20}O_{20}H^{+}_{1}$	-0.50	2-Internal
2081.44294	1	$C_{105}H_{189}N_{21}O_{21}H^{+}_{1}$	-0.49	2-Internal
2180.51554	1	$C_{110}H_{198}N_{22}O_{22}H^{+}_{1}$	1.45	2-Internal
2378.64907	1	$C_{120}H_{216}N_{24}O_{24}H^{+}_{1}$	-0.06	2-Internal
2477.72073	1	$C_{125}H_{225}N_{25}O_{25}H^{+}_{1}$	1.25	2-Internal
324.22818	1	$C_{17}H_{29}N_{3}O_{3}H^{+}_{1}$	0.04	3-Internal
423.29666	1	$C_{22}H_{38}N_{4}O_{4}H^{+}_{1}$	0.18	3-Internal
522.36515	1	$C_{27}H_{47}N_{5}O_{5}H^{+}_{1}$	0.29	3-Internal
621.4336	1	$C_{32}H_{56}N_{6}O_{6}H^{+}_{1}$	0.31	3-Internal
212.152	2	$C_{22}H_{38}N_{4}O_{4}H^{+}_{2}$	0.33	3-Internal
261.68619	2	$C_{27}H_{47}N_{5}O_{5}H^{+}_{2}$	0.21	3-Internal
360.75464	2	$C_{37}H_{65}N_{7}O_{7}H^{+}_{2}$	0.25	3-Internal
410.28887	2	$C_{42}H_{74}N_{8}O_{8}H^{+}_{2}$	0.28	3-Internal
459.82306	2	$C_{47}H_{83}N_{9}O_{9}H^{+}_{2}$	0.21	3-Internal
509.35726	2	$C_{52}H_{92}N_{10}O_{10}H^{+}_{2}$	0.17	3-Internal
558.89154	2	$C_{57}H_{101}N_{11}O_{11}H^{+}_{2}$	0.29	3-Internal
608.42572	2	$C_{62}H_{110}N_{12}O_{12}H^{+}_{2}$	0.22	3-Internal
657.95995	2	$C_{67}H_{119}N_{13}O_{13}H^{+}_{2}$	0.24	3-Internal
707.49425	2	$C_{72}H_{128}N_{14}O_{14}H^{+}_{2}$	0.35	3-Internal
757.02847	2	$C_{77}H_{137}N_{15}O_{15}H^{+}_{2}$	0.35	3-Internal
806.56262	2	$C_{82}H_{146}N_{16}O_{16}H^{+}_{2}$	0.26	3-Internal
856.09693	2	$C_{87}H_{155}N_{17}O_{17}H^{+}_{2}$	0.36	3-Internal
905.63081	2	$C_{92}H_{164}N_{18}O_{18}H^{+}_{2}$	-0.02	3-Internal
955.16538	2	$C_{97}H_{173}N_{19}O_{19}H^{+}_{2}$	0.36	3-Internal
1054.23329	2	$C_{107}H_{191}N_{21}O_{21}H^{+}_{2}$	-0.15	3-Internal
169.13359	1	$C_{9}H_{16}N_{2}O_{1}H^{+}_{1}$	0.30	4-Internal
268.20198	1	$C_{14}H_{25}N_{3}O_{2}H^{+}_{1}$	0.10	4-Internal
367.27039	1	$C_{19}H_{34}N_{4}O_{3}H^{+}_{1}$	0.06	4-Internal
466.33888	1	$C_{24}H_{43}N_{5}O_{4}H^{+}_{1}$	0.21	4-Internal
565.4073	1	$C_{29}H_{52}N_{6}O_{5}H^{+}_{1}$	0.19	4-Internal
664.47588	1	$C_{34}H_{61}N_{7}O_{6}H^{+}_{1}$	0.41	4-Internal
763.54368	1	$C_{39}H_{70}N_{8}O_{7}H^{+}_{1}$	-0.45	4-Internal
862.61313	1	$C_{44}H_{79}N_{9}O_{8}H^{+}_{1}$	0.80	4-Internal
961.68164	1	$C_{49}H_{88}N_{10}O_{9}H^{+}_{1}$	0.82	4-Internal
233.67305	2	$C_{24}H_{43}N_{5}O_{4}H^{+}_{2}$	0.09	4-Internal
283.20726	2	$C_{29}H_{52}N_{6}O_{5}H^{+}_{2}$	0.09	4-Internal
332.74147	2	$C_{34}H_{61}N_{7}O_{6}H^{+}_{2}$	0.08	4-Internal
382.27572	2	$C_{39}H_{70}N_{8}O_{7}H^{+}_{2}$	0.18	4-Internal
431.81	2	$C_{44}H_{79}N_{9}O_{8}H^{+}_{2}$	0.33	4-Internal
481.34428	2	$C_{49}H_{88}N_{10}O_{9}H^{+}_{2}$	0.45	4-Internal
530.87844	2	$C_{54}H_{97}N_{11}O_{10}H^{+}_{2}$	0.32	4-Internal
580.41265	2	$C_{59}H_{106}N_{12}O_{11}H^{+}_{2}$	0.30	4-Internal
629.94689	2	$C_{64}H_{115}N_{13}O_{12}H^{+}_{2}$	0.33	4-Internal
679.48122	2	$C_{69}H_{124}N_{14}O_{13}H^{+}_{2}$	0.48	4-Internal
729.01551	2	$C_{74}H_{133}N_{15}O_{14}H^{+}_{2}$	0.56	4-Internal
828.08369	2	$C_{84}H_{151}N_{17}O_{16}H^{+}_{2}$	0.21	4-Internal

877.61857	2	$C_{89}H_{160}N_{18}O_{17}H^{+}_{2}$	0.97	4-Internal
927.15202	2	$C_{94}H_{169}N_{19}O_{18}H^{+}_{2}$	0.10	4-Internal
173.12852	1	$C_{8}H_{16}N_{2}O_{2}H^{+}_{1}$	0.38	5-Internal
272.19691	1	$C_{13}H_{25}N_{3}O_{3}H^{+}_{1}$	0.15	5-Internal
371.26533	1	$C_{18}H_{34}N_{4}O_{4}H^{+}_{1}$	0.13	5-Internal
470.33379	1	$C_{23}H_{43}N_{5}O_{5}H^{+}_{1}$	0.20	5-Internal
569.40226	1	$C_{28}H_{52}N_{6}O_{6}H^{+}_{1}$	0.26	5-Internal
668.47076	1	$C_{33}H_{61}N_{7}O_{7}H^{+}_{1}$	0.35	5-Internal
767.53909	1	$C_{38}H_{70}N_{8}O_{8}H^{+}_{1}$	0.20	5-Internal
866.60757	1	$C_{43}H_{79}N_{9}O_{9}H^{+}_{1}$	0.25	5-Internal
965.67627	1	$C_{48}H_{88}N_{10}O_{10}H^{+}_{1}$	0.52	5-Internal
1064.74473	1	$C_{53}H_{97}N_{11}O_{11}H^{+}_{1}$	0.52	5-Internal
532.87586	2	$C_{53}H_{97}N_{11}O_{11}H^{+}_{2}$	0.25	5-Internal
631.94434	2	$C_{63}H_{115}N_{13}O_{13}H^{+}_{2}$	0.31	5-Internal
681.47848	2	$C_{68}H_{124}N_{14}O_{14}H^{+}_{2}$	0.19	5-Internal
731.01289	2	$C_{73}H_{133}N_{15}O_{15}H^{+}_{2}$	0.46	5-Internal
780.54696	2	$C_{78}H_{142}N_{16}O_{16}H^{+}_{2}$	0.25	5-Internal
830.08143	2	$C_{83}H_{151}N_{17}O_{17}H^{+}_{2}$	0.55	5-Internal
879.61534	2	$C_{88}H_{160}N_{18}O_{18}H^{+}_{2}$	0.19	5-Internal
929.14987	2	$C_{93}H_{169}N_{19}O_{19}H^{+}_{2}$	0.52	5-Internal
978.68392	2	$C_{98}H_{178}N_{20}O_{20}H^{+}_{2}$	0.34	5-Internal
1028.21807	2	$C_{103}H_{187}N_{21}O_{21}H^{+}_{2}$	0.27	5-Internal
1077.75266	2	$C_{108}H_{196}N_{22}O_{22}H^{+}_{2}$	0.61	5-Internal
1127.28607	2	$C_{113}H_{205}N_{23}O_{23}H^{+}_{2}$	-0.13	5-Internal
1176.82132	2	$C_{118}H_{214}N_{24}O_{24}H^{+}_{2}$	0.77	5-Internal
1374.95844	2	$C_{138}H_{250}N_{28}O_{28}H^{+}_{2}$	0.87	5-Internal
1424.49151	2	$C_{143}H_{259}N_{29}O_{29}H^{+}_{2}$	0.04	5-Internal
1523.5609	2	$C_{153}H_{277}N_{31}O_{31}H^{+}_{2}$	0.68	5-Internal
1573.09503	2	$C_{158}H_{286}N_{32}O_{32}H^{+}_{2}$	0.61	5-Internal
1622.62715	2	$C_{163}H_{295}N_{33}O_{33}H^{+}_{2}$	-0.70	5-Internal
1672.16126	2	$C_{168}H_{304}N_{34}O_{34}H^{+}_{2}$	-0.73	5-Internal
1721.69778	2	$C_{173}H_{313}N_{35}O_{35}H^{+}_{2}$	0.63	5-Internal
1771.23576	2	$C_{178}H_{322}N_{36}O_{36}H^{+}_{2}$	2.74	5-Internal
619.76899	3	$C_{93}H_{169}N_{19}O_{19}H^{+}_{3}$	0.50	5-Internal
652.79165	3	$C_{98}H_{178}N_{20}O_{20}H^{+}_{3}$	0.25	5-Internal
685.81454	3	$C_{103}H_{187}N_{21}O_{21}H^{+}_{3}$	0.36	5-Internal
718.83742	3	$C_{108}H_{196}N_{22}O_{22}H^{+}_{3}$	0.45	5-Internal
751.86018	3	$C_{113}H_{205}N_{23}O_{23}H^{+}_{3}$	0.37	5-Internal
784.88316	3	$C_{118}H_{214}N_{24}O_{24}H^{+}_{3}$	0.58	5-Internal
817.90572	3	$C_{123}H_{223}N_{25}O_{25}H^{+}_{3}$	0.26	5-Internal
850.9286	3	$C_{128}H_{232}N_{26}O_{26}H^{+}_{3}$	0.34	5-Internal
883.95126	3	$C_{133}H_{241}N_{27}O_{27}H^{+}_{3}$	0.16	5-Internal
916.97421	3	$C_{138}H_{250}N_{28}O_{28}H^{+}_{3}$	0.31	5-Internal
949.99695	3	$C_{143}H_{259}N_{29}O_{29}H^{+}_{3}$	0.23	5-Internal
983.01956	3	$C_{148}H_{268}N_{30}O_{30}H^{+}_{3}$	0.03	5-Internal
1016.04223	3	$C_{153}H_{277}N_{31}O_{31}H^{+}_{3}$	-0.11	5-Internal
1049.06543	3	$C_{158}H_{286}N_{32}O_{32}H^{+}_{3}$	0.27	5-Internal
1082.08805	3	$C_{163}H_{295}N_{33}O_{33}H^{+}_{3}$	0.10	5-Internal
1115.111	3	$C_{168}H_{304}N_{34}O_{34}H^{+}_{3}$	0.22	5-Internal

1148.13416	3	$C_{173}H_{313}N_{35}O_{35}H^{+}_{3}$	0.53	5-Internal
1181.15657	3	$C_{178}H_{322}N_{36}O_{36}H^{+}_{3}$	0.18	5-Internal
1280.22556	3	$C_{193}H_{349}N_{39}O_{39}H^{+}_{3}$	0.61	5-Internal
1313.24734	3	$C_{198}H_{358}N_{40}O_{40}H^{+}_{3}$	-0.18	5-Internal
1346.26874	3	$C_{203}H_{367}N_{41}O_{41}H^{+}_{3}$	-1.22	5-Internal
1379.29238	3	$C_{208}H_{376}N_{42}O_{42}H^{+}_{3}$	-0.59	5-Internal
1412.317	3	$C_{213}H_{385}N_{43}O_{43}H^{+}_{3}$	0.71	5-Internal
1445.33873	3	$C_{218}H_{394}N_{44}O_{44}H^{+}_{3}$	-0.05	5-Internal
1478.36196	3	$C_{223}H_{403}N_{45}O_{45}H^{+}_{3}$	0.24	5-Internal
1544.40792	3	$C_{233}H_{421}N_{47}O_{47}H^{+}_{3}$	0.46	5-Internal
286.21255	1	$C_{14}H_{27}N_{3}O_{3}H^{+}_{1}$	0.11	6-Internal
385.28096	1	$C_{19}H_{36}N_{4}O_{4}H^{+}_{1}$	0.07	6-Internal
484.34946	1	$C_{24}H_{45}N_{5}O_{5}H^{+}_{1}$	0.24	6-Internal
583.41736	1	$C_{29}H_{54}N_{6}O_{6}H^{+}_{1}$	-0.69	6-Internal
682.48637	1	$C_{34}H_{63}N_{7}O_{7}H^{+}_{1}$	0.29	6-Internal
781.55521	1	$C_{39}H_{72}N_{8}O_{8}H^{+}_{1}$	0.80	6-Internal
880.62417	1	$C_{44}H_{81}N_{9}O_{9}H^{+}_{1}$	1.33	6-Internal
440.81514	2	$C_{44}H_{81}N_{9}O_{9}H^{+}_{2}$	0.00	6-Internal
638.9522	2	$C_{64}H_{117}N_{13}O_{13}H^{+}_{2}$	0.36	6-Internal
688.48676	2	$C_{69}H_{126}N_{14}O_{14}H^{+}_{2}$	0.85	6-Internal
738.01965	2	$C_{74}H_{135}N_{15}O_{15}H^{+}_{2}$	-0.99	6-Internal
787.55518	2	$C_{79}H_{144}N_{16}O_{16}H^{+}_{2}$	0.75	6-Internal
886.62334	2	$C_{89}H_{162}N_{18}O_{18}H^{+}_{2}$	0.38	6-Internal
936.15274	2	$C_{94}H_{171}N_{19}O_{19}H^{+}_{2}$	-4.77	6-Internal
381.27382	3	$C_{59}H_{104}N_{12}O_{10}H^{+}_{3}$	-0.18	Internal
414.29626	3	$C_{64}H_{113}N_{13}O_{11}H^{+}_{3}$	-1.05	Internal
447.31983	3	$C_{69}H_{122}N_{14}O_{12}H^{+}_{3}$	0.74	Internal
546.38811	3	$C_{84}H_{149}N_{17}O_{15}H^{+}_{3}$	0.36	Internal
579.4108	3	$C_{89}H_{158}N_{18}O_{16}H^{+}_{3}$	0.14	Internal
612.433	3	$C_{94}H_{167}N_{19}O_{17}H^{+}_{3}$	-0.85	Internal
645.45689	3	$C_{99}H_{176}N_{20}O_{18}H^{+}_{3}$	0.87	Internal
678.47932	3	$C_{104}H_{185}N_{21}O_{19}H^{+}_{3}$	0.28	Internal
711.50123	3	$C_{109}H_{194}N_{22}O_{20}H^{+}_{3}$	-0.99	Internal
744.52399	3	$C_{114}H_{203}N_{23}O_{21}H^{+}_{3}$	-1.01	Internal
810.56953	3	$C_{124}H_{221}N_{25}O_{23}H^{+}_{3}$	-1.01	Internal
843.59231	3	$C_{129}H_{230}N_{26}O_{24}H^{+}_{3}$	-1.00	Internal
876.61577	3	$C_{134}H_{239}N_{27}O_{25}H^{+}_{3}$	-0.22	Internal
909.63891	3	$C_{139}H_{248}N_{28}O_{26}H^{+}_{3}$	0.16	Internal
942.66016	3	$C_{144}H_{257}N_{29}O_{27}H^{+}_{3}$	-1.49	Internal
975.68437	3	$C_{149}H_{266}N_{30}O_{28}H^{+}_{3}$	0.00	Internal
1008.70532	3	$C_{154}H_{275}N_{31}O_{29}H^{+}_{3}$	-1.84	Internal
1041.72842	3	$C_{159}H_{284}N_{32}O_{30}H^{+}_{3}$	-1.50	Internal
1074.7523	3	$C_{164}H_{293}N_{33}O_{31}H^{+}_{3}$	-0.45	Internal
644.95166	2	$C_{65}H_{117}N_{13}O_{13}H^{+}_{2}$	-0.48	Internal
694.48643	2	$C_{70}H_{126}N_{14}O_{14}H^{+}_{2}$	0.37	Internal
793.55484	2	$C_{80}H_{144}N_{16}O_{16}H^{+}_{2}$	0.32	Internal
843.08937	2	$C_{85}H_{153}N_{17}O_{17}H^{+}_{2}$	0.68	Internal
942.15772	2	$C_{95}H_{171}N_{19}O_{19}H^{+}_{2}$	0.54	Internal
991.69191	2	$C_{100}H_{180}N_{20}O_{20}H^{+}_{2}$	0.50	Internal

1041.22599	2	$C_{105}H_{189}N_{21}O_{21}H^{+}_{2}$	0.35	Internal
1090.76048	2	$C_{110}H_{198}N_{22}O_{22}H^{+}_{2}$	0.60	Internal
1140.29463	2	$C_{115}H_{207}N_{23}O_{23}H^{+}_{2}$	0.52	Internal
1189.82824	2	$C_{120}H_{216}N_{24}O_{24}H^{+}_{2}$	0.00	Internal
1288.89721	2	$C_{130}H_{234}N_{26}O_{26}H^{+}_{2}$	0.43	Internal
1338.43102	2	$C_{135}H_{243}N_{27}O_{27}H^{+}_{2}$	0.12	Internal
1437.49851	2	$C_{145}H_{261}N_{29}O_{29}H^{+}_{2}$	-0.53	Internal
		Average	0.53	

Fragment	Initial fragment mass	Error (ppm)	Ref Code (table above)
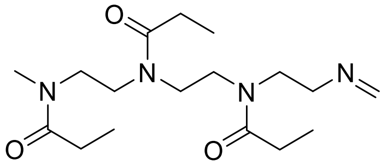	341.25478	0.2	1
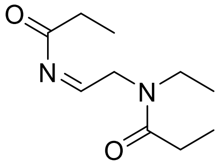	199.14414	0.2	2
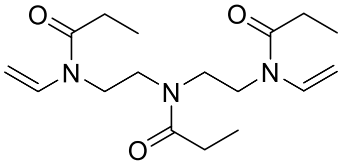	324.22818	0.0	3
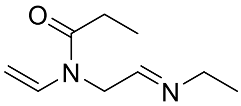	169.13359	0.3	4
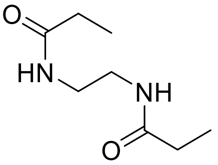	173.12852	0.4	5
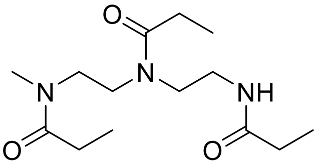	286.21255	0.1	6

(1) Theisen, A.; Wootton, C. A.; Haris, A.; Morgan, T. E.; Lam, Y. P. Y.; Barrow, M. P.; O'Connor, P. B. Enhancing Biomolecule Analysis and 2DMS Experiments by Implementation of (Activated Ion) 193 nm UVPD on a FT-ICR Mass Spectrometer. *Anal Chem* **2022**, *94* (45), 15631-15638
